# A Review of Microelectronic Systems and Circuit Techniques for Electrical Neural Recording Aimed at Closed-Loop Epilepsy Control

**DOI:** 10.3390/s20195716

**Published:** 2020-10-08

**Authors:** Reza Ranjandish, Alexandre Schmid

**Affiliations:** 1 Department of Information Technology and Electrical Engineering, ETH Zürich, CH-8092 Zürich, Switzerland; rranjandish@ethz.ch; 2 Institute of Electrical Engineering, EPF Lausanne, CH-1015 Lausanne, Switzerland

**Keywords:** epilepsy, seizure, multichannel neural recording, feature extraction, closed-loop neurostimulator, low-power, low-noise amplifier, implantable medical device

## Abstract

Closed-loop implantable electronics offer a new trend in therapeutic systems aimed at controlling some neurological diseases such as epilepsy. Seizures are detected and electrical stimulation applied to the brain or groups of nerves. To this aim, the signal recording chain must be very carefully designed so as to operate in low-power and low-latency, while enhancing the probability of correct event detection. This paper reviews the electrical characteristics of the target brain signals pertaining to epilepsy detection. Commercial systems are presented and discussed. Finally, the major blocks of the signal acquisition chain are presented with a focus on the circuit architecture and a careful attention to solutions to issues related to data acquisition from multi-channel arrays of cortical sensors.

## 1. Introduction

Over a long period of time of being constrained to experiment, bioelectricity has matured into the fundaments of bio-electronic interfaces, paving the way to new therapeutic systems in recording or stimulation modes of operation. Implantable electronic medical devices (IEMDs) have emerged in recent years, based on the success of early medical applications of bioelectricity, including for instance external electro-cardiograms (ECG), recording or external heart defibrillators (stimulation), as well as based on the advancements of low-power integrated microelectronics.

Commercial IEMD products are multiple and include, for instance, heart monitoring systems such as the CardioMEMS HF System provided by Abbott Laboratories for heart failure detection [1], heart pacemakers such as the Momentum X4 provided by Boston Scientific that applies electric pulses to the heart muscles to regulate its contractions [2]. Additional successful IEMDs include cochlear implants aimed at restoring hearing capability, pain control devices, deep-brain implantable systems aimed at controlling and eluding tremors due to Parkinson’s disease, and recently, retina implants aimed at restoring lost vision by electrical stimulation of remaining healthy retina tissues.

This paper reviews microelectronic techniques aimed at epilepsy control in a closed loop. Epilepsy is briefly discussed from an engineering perspective in Section 1.1, i.e., with a focus on the conditions and parameters that are specific and relevant to electrical neuromodulation. The architecture of epilepsy-control implantable systems is presented in Section 2. Classical electrodes are presented along with a discussion of electrical recording and stimulation of the brain. Commercial systems aimed at epilepsy control are reviewed, as implantable and external systems, as open and closed-loop systems and discussed in terms of their operational principles, advantages and drawbacks in Section 3. Finally, analog front-end amplifiers are reviewed in terms of classical topologies aimed at single, and multi-channel recording from the brain in Section 4. The major blocks in the recording chain that are specific to epilepsy control are reviewed and discussed. A detailed review of the stimulation chain is deliberately not considered in this review.

### 1.1. Engineering Overview of Epilepsy, Seizures and Treatment

Neurological disorders are defined as diseases that affect the nervous system, including the central and peripheral nervous systems. Among multiple different neurological disorders, several diseases are prevalent such as amyotrophic lateral sclerosis, brain tumors, epilepsy, Parkinson, etc. Epilepsy consists of the recurrence of a phenomenon called a seizure. A seizure occurs when the brain produces a brief abnormal and uncontrollable electrical discharge. Clinically, according to the International League Against Epilepsy (ILAE) official report [3], epilepsy is considered to be a disease of the brain defined by any following conditions:

- At least two unprovoked (or reflex) seizures occurring >24 h apart.
- one unprovoked (or reflex) seizure and a probability of further seizures similar to the general recurrence risk (at least 60%) after two unprovoked seizures, occurring over the next 10 years.
- diagnosis of an epilepsy syndrome.

#### 1.1.1. Phases of a Seizure

Seizures often proceed over four consecutive phases; prodromal, auras, ictal and postictal. Some patients experience a prodromal stage which mostly involves emotional signals. This stage may develop over hours or even days before the start of the seizure. The second phase which is called aura occurs immediately before a seizure onset and may last for a few seconds. During aura, patients may experience déjà vu, jamais vu, headaches and other symptoms. The third phase, which is called ictal, covers the period over which a seizure extends. This period is correlated with the electrical seizure activity in the brain. The fourth phase, which is called postictal is a period of recovery from a seizure. The postictal stage is different among the patients and may last from a few minutes to a few hours. The recovery period is determined by the type of seizure (Section 1.1.2) as well as the part(s) of the brain involved in the seizure.

#### 1.1.2. Seizure Classification

Understanding and diagnosis of the seizure type bears a significant implication on the daily life of an epileptic patient. The type of seizure determines whether a patient can safely perform some common daily-life tasks, including driving, and sports. Natural tasks with potential high social impediment are affected by seizures, including walking and communicating. In addition, the type of seizure has a tremendous impact on understanding which medication is suitable for the treatment or which medication may potentially be harmful [4].

**Figure 1:**
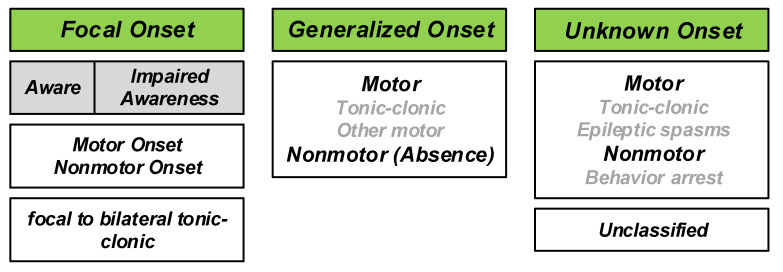
The new classification of seizure types based on the International League Against Epilepsy (ILAE) report (Reprinted with permission of Wiley Periodicals, Inc. © 2017 International League Against Epilepsy) [7].

Describing the different types of seizures was initiated in the times of Hippocrates. In 1964, Gastaut proposed a new and modern classification of seizures [5]. The traditional classification of seizures is based on anatomy comprising temporal, frontal, parietal, occipital, diencephalic, or brainstem seizures. However, the understanding of the mechanism of seizures has evolved thanks to modern research. In 1981, an ILAE commission classified seizures into partial and generalized-onset, simple and complex partial seizures, as well as various specific generalized types [6,7]. This classification was established from the study of hundreds of video–electroencephalography (EEG) recordings of seizures. This latter classification is the main reference to date, with some terminology revisions [8,9]. One of the newest classifications is the 2017 classification proposed by ILAE [7]. The overview of the new classification is shown in Figure 1. With respect to the 1981 classification, the 2017 classification significantly reduces the number of unclassifiable cases. The combination of motor/non-motor and awareness level features provides better flexibility and detailed seizure description [10]. The new classification is based on several important facts expressed as follows [4]:

- The onset or beginning of a seizure;
- a person’s level of awareness during a seizure, and
- whether body movements occur during a seizure.

Based on the 2017 classification, three major groups of seizures are observed, including generalized onset seizures, focal onset seizures and unknown onset seizures. Generalized seizures affect both sides of the brain or group of cells located over both sides of the brain, simultaneously. Focal seizures (Partial seizures in the 1981 classification) start in one area or group of cells in the brain. Unknown onset seizures is the term used when the location of the seizure onset is unknown.

#### 1.1.3. Statistics

According to World Health Organization (WHO) [11], approximately 50 million people worldwide suffer from epilepsy. 80% of them live in low and middle-income countries. Among them 75% do not receive proper treatment. The estimated proportion of the general population with active epilepsy (continuing seizures or with the need for treatment) is between 4 and 10 per 1000 people. However, this number is much higher in low and middle-income countries, i.e., between 7 and 14 per 1000 people.

Each year, 2.4 million people are diagnosed with epilepsy [11]. The estimated proportion of new cases with epilepsy in high-income countries is between 30 and 50 per 100,000 people. This rate doubles in low and middle-income countries.

#### 1.1.4. Epilepsy Treatment

Several studies have explored the reason for the initiation of seizures [12,13]. The importance of finding the reasons and origin lies in the correct diagnosis and treatment of epilepsy. No medical treatment can be safely provided without having knowledge of the different sources that cause epilepsy. The development of modern medical devices and implantable systems participate in improving the understanding of the origins of epilepsy.

The treatment of epilepsy is composed of three levels. The first level is the treatment with medications. Several anti-epileptic drugs (AEDs) are available for epilepsy patients. There are approximately 25 different AEDs suitable for controlling seizures; different AEDs are suitable for different seizure types. The benefits of AEDs consist of the reduction of stopping seizures and risk of accidents. A first-line AED is an AED that is tried first in the therapy. The AEDs added to the first-line AED are called second-line AEDs. The success rate of the third medication delivered after trying two different medications is approximately 5%.

Epilepsy is defined as a drug-resistant epilepsy when a patient has unsuccessfully tried two different anti-seizure medications. The second level of epilepsy treatment is lobectomy. Lobectomy is the removal of the part of the brain that is responsible for seizure initiation, if the region is uniquely detectable, and if it is not in a sensitive region of the brain. Before lobectomy, surgeons must find the precise location of the brain from which the seizures start. Following modern medical procedures, seizures can be identified and categorized using three Tesla magnetic resonance imaging (3T MRI), video-EEG, single-photon emission computed tomography (SPECT), magnetoencephalography (MEG) scan and positron emission tomography (PET) scan as prerequisites of further treatment steps. A resection surgery would be prescribed in cases of a focal seizure and if a single location can be identified [14]. In a resection surgery, a part of the skull is temporarily removed by craniotomy and the part of the brain which is engaged in the seizure initiation is removed. Laser thermal ablation is another way to stop the seizures, which is a minimally invasive method. This method is performed in the MRI in real time. In this surgery, during an MRI scan, the part of the brain that is engaged in the seizure initiation is heated up and destroyed, while local temperature is monitored using the MRI scanner. This method can only be employed in limited areas of the brain.

In order to precisely detect the part of the brain that is a target of resection surgery or laser thermal ablation, intracranial monitoring is necessary, which is a diagnostic surgery. Intracranial monitoring is carried out in two different ways, including recording using subdural grids and strips or stereo-EEG using SEEG electrodes.

Patients responding to prevalent cures like medications and surgeries are approximately 70%, while approximately 30% of the patients are untreated or poorly treated because of the following reasons:

- Seizures diffuse over an excessively large area;
- seizures occur in sensitive areas of eloquent cortex that may not be surgically treated;
- seizures have multiple foci (multifocal seizures) which are thus difficult to individual localization and in practice impossible to surgically treat;
- surgery may not be tolerable due to specific medical conditions.

Implantable electrical stimulators offer an alternative therapy to untreated or poorly treated patients whose seizures are not controlled using medication or surgery. These stimulator systems either operate in an open-loop or closed-loop mode. IEMDs operating in open-loop stimulation only consist of electrical stimulators. In contrast, closed-loop stimulators record neural signals from the brain and detect the seizure onsets. Upon seizure detection, closed-loop stimulators trigger electrical stimulation. Commercial stimulators are reviewed in Section 3.

Intracranial Pressure (ICP) is reported to influence drug-resistant epilepsy in some cases. In [15], a 23-year old patient was reported as the case study. In this study, an increase in ICP is shown to augment the efficiency of anti-seizure medications. The epileptic patient had undergone a shunt surgery in childhood to decrease the ICP. The patient had uncontrolled seizures in spite of three years of pharmacotherapy. However, the uncontrolled seizures suddenly stopped after shunt removal.

## 2. Introduction to Epilepsy Control Using Implantable Microelectronic Systems

**Figure 2:**
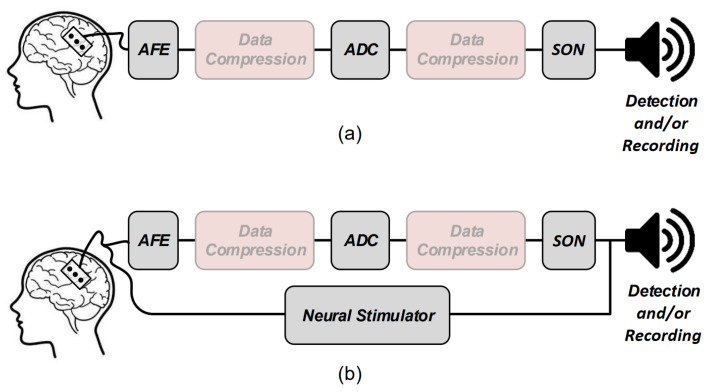
General overview of (**a**) a low-power seizure detection, and (**b**) a low-power seizure control systems.

Several electronic building blocks are essential to design and implement a low-power seizure detection system. These blocks are also some of the main building blocks of an epilepsy control system. Figure 2 shows the general overview of a low-power seizure detection (Figure 2a) and epilepsy control (Figure 2b) system. Both systems have common essential building blocks which are shown with gray boxes including the analog front-end (AFE), analog-to-digital converter (ADC), and seizure onset detector (SON). The Epilepsy control system has an additional essential building block which is the electrical neural stimulator aimed at suppressing or modulating the seizure electrical activity. In addition, data compression may be applied prior to feeding the data to the SON to lower the power consumption of the systems. Data compression can be done either in analog or digital domains.

In the following, the operation principles of some of these blocks are briefly reviewed, including the neural amplifiers, compressive sensing and feature extractors inside the SON block.

### 2.1. Electrical Stimulation

The historical developments of electrical stimulators are considered to begin in the European post-Middle Ages, especially from the 16th century when a Dutch scientist, Jan Swammerdam, performed the first experiment on the muscle of a dissected frog [16]. He realized that the severed muscle of a frog is contracted by irritation. The idea of contracting a muscle by a stimuli had an important impact on neuroscience by demonstrating the fact that the observed behavior is based on the stimuli. More than one hundred years after Swammerdam’s first experiment, on 6 November 1787, Luigi Galvani realized that a frog muscle can be contracted by placing an iron wire to the muscle and a copper wire to the nerve during a random experiment. He observed that an animal body performs convulsive movements when electricity is applied to it. The work of Galvani inspired Alessandro Volta to invent the voltaic pile in 1799. Using a voltaic pile, Luigi Rolando performed the first cortical stimulation experiment on an animal cortex in 1809. Indeed, Rolando is well-known for his pioneering research on brain localization of function. In 1825, J.-B. Sarlandiere published an extensive study on the benefits of electricity for pain relief by applying electricity to acupuncture needles. Gustav Fritsch and Eduard Hitzing published an article in 1870, showing that the stimulation of some specific part of the cortex leads to muscle contraction in dogs. Robert Bartholow, who was an American physician, was the first to report the findings of a study on electrical stimulation applied to the cerebral cortex of an awake human in 1874. All of these studies led to the design and introduction of different types of modern electrical stimulators and implantable stimulation devices.

Medical devices for electrical stimulation can be considered to belong to two classes, including implantable electrical stimulators and external electrical stimulators. External electrical stimulators are also categorized as transcranial and transcutaneous electrical stimulators. Some of the well-known external electrical stimulation methods include transcranial Alternating Current Stimulation (tACS), transcranial Direct Current Stimulation (tDCS), transcutaneous Trigeminal Nerve Stimulation (tTNS), transcutaneous Vagus Nerve Stimulation (tVNS) and transcutaneous Electric Nerve Stimulation (tENS). Each type of external electrical stimulator is developed, aiming at a specific application and target. For example, tTNS stimulators are currently commercialized for the relief and prevention of headaches. A commercialized tTNS is designed by Cefaly Technology. Implantable stimulators including deep-brain stimulation (DBS), retinal implants, pacemakers, cochlear implants and functional electrical stimulation (FES) are employed to assist or restore the functionality of organs that are not properly functioning. Electrical stimulators operate on the principle of initiating an action potential (AP) upon the transfer of electrical charge into excitable tissue. Electrical stimulators operate in three distinct modes, namely voltage-mode stimulation, current-mode stimulation and charge-mode stimulation.

Safety issues of biological cells impose a strict condition on the electrical pulses, i.e., a biphasic stimulation is necessary to prevent tissue damage and any long-term effects such as pH shift (during the usage of IEMDs) and erosion of the electrodes. Biphasic stimulation consists of a cathodic phase followed by an anodic phase. During the cathodic phase, the cell membrane is depolarized. Then, the anodic phase neutralizes the charge which has been injected during the cathodic phase. For exerting a safe stimulation, the voltage across the electrode must be constraint within a specific window. In addition, to block any direct current passing through the tissue, a large off-chip capacitor, namely a blocking capacitor, is placed in series with the stimulation electrode. This capacitor blocks the flow of any dc current through the tissue in case of semiconductor failure which makes the stimulator fail-safe. However, in multichannel stimulators such as retinal or cochlear implants, a large silicon area cannot be allocated to the large blocking capacitors. Hence, various circuits are proposed in the literature to substitute blocking capacitors with active circuits and to reduce the overall size of the stimulation system.

### 2.2. Physiological Signal Recording

**Figure 3:**
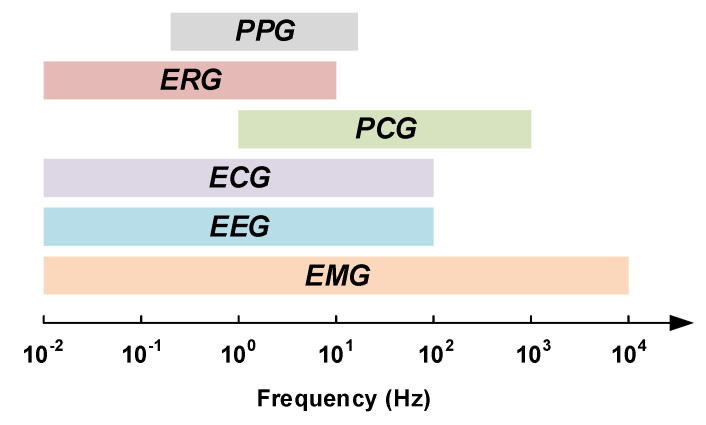
Bandwidths of some vital physiological signals (modified from [20]).

The current healthcare systems are expected to deal with two major issues including chronic diseases and global population aging [17,18,19]. Early detection, as well as timely treatment of diseases, require monitoring systems that allow physicians to closely monitor the physiological signals of their patients. Acquiring physiological signals requires robust, light-weight and low-power wearable or implantable electronic medical devices. There are several important physiological signals that provide vital information of the human body such as electromyogram signals (EMG), electroencephalogram signals (EEG), electrocardiogram signals (ECG), phonocardiogram signals (PCG), electroretinography signals (ERG) and photoplethysmograms (PPG). Each of these signals has its own electrical characteristics, i.e., they have specific bandwidth and maximum amplitude. The bandwidths of some of these signals are depicted in Figure 3.

Although epilepsy can be detected from different physiological signals, the most important signal to predict or to detect a seizure onset are neural signals recorded from the brain. The following Subsection describes the characteristics of different types of neural signals that can be recorded from different types of electrodes.

#### Neural Signal Recording

Historically, electrophysiology is based on the discovery of Italian scientist Galvani (1737–1798). Galvani realized that the tissues of frog muscles exhibit electrical potential. Studies on the living tissues continued until Hans Berger (1873–1941) discovered the human electroencephalogram (EEG). Berger could record the first human EEG signal using a Siemens double-coil galvanometer in 1924. When Berger’s work was confirmed, EEG started to be used in clinical trials. Although vacuum tubes amplifiers were available since 1906, it took long time before they were used in the recording of neural activities. A group of researchers including Fredrick Gibbs (1903–1992), Hallowell Davis (1896–1992) and WG Lennox (1884–1960) with an EEG technician, namely Erna Gibbs (1906–1987), demonstrated EEG signals corresponding to a clinical absence attack [21] in 1935. They showed that the inter-ictal signals corresponding to an absence seizure attack have a specific signature of three spikes per second. Shortly after, in 1936, F. Gibbs demonstrated the importance of EEG in diagnosis and localization of epileptic seizures. Collaboration between F. Gibbs and Albert Grass, a MIT graduate, resulted in the development of EEG recording systems which smoothed the way for Grass Instrument Company. Grass Instrument Company was founded by A. Grass and his wife, Ellen, in 1945 and was acquired by Astro-Med Inc. in 1994, two years after the death of A. Grass. In 1946, the American EEG society (AEEGS) was founded and a year after, in 1947, the first annual meeting of the American EEG society was held in Atlantic City, NJ on 13–15 June [22]. In 1950s, William Grey Walter (1910–1977) developed the first EEG topography machine which can show a map of brain activity. During this decade, Wilder Penfield (1891–1976) and Herbert Jasper (1906–1999), neurosurgeons at the Montreal Neurological Institute, developed electro-corticography (ECoG) as a part of a surgical procedure for treating patients with severe epilepsy. The brain activity is recorded from the cerebral cortex using ECoG. It is shown that recording using ECoG has higher spacial resolution than EEG. Thus, this method is preferred for finding the regions of the cortex that generates epileptic seizures. Until now, recording brain waves has evolved thanks to the improvements in the technology of the electrodes. Nowadays, recording the activity of a single neuron is possible.

Decoding the functional operation of the brain requires recording of the electrical activity of neurons of the central nervous system [23]. Understanding and diagnosing neurological disorders such as epilepsy are based on the recording of the brain electrical activities. Furthermore, neural recording is a major module of brain–machine interfaces and neuroprosthetic technologies that aim at aiding paralyzed patients [24,25,26]. The demand for technologies that empower the neuroscientist and clinicians to observe the electrical activity of a large population of neurons in the brain has increased in the last decade. Extremely complex circuit solutions are needed to simultaneously monitor a large population of neurons in the brain that exceeds hundreds of cites in some applications [27], Simultaneous recording of the neural activities requires low-noise, low-power and area-efficient amplifiers. Good gain matching over the various channels and low crosstalk over the channels are other criteria.

**Figure 4:**
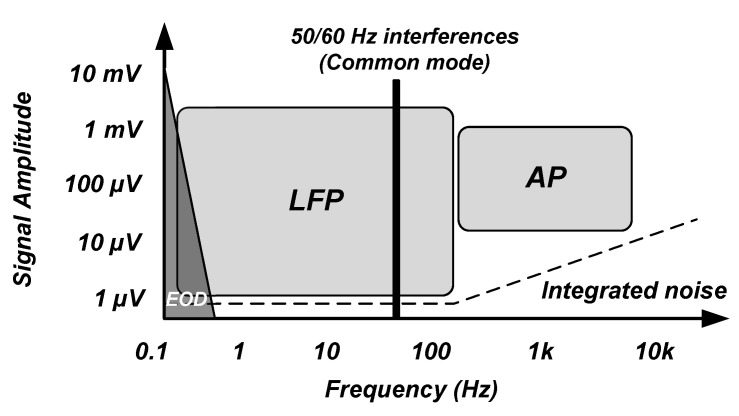
Signals characteristics in neural recording systems.

The neural signals of interest for the recording of the brain activity using implantable devices have a frequency band of 1 Hz to 5 KHz [28]. As shown in Figure 4 [25,28,29,30], these signals consist of local field potentials (LFPs) and action potentials (APs) which are shown to include biomarkers that are useful for diagnosis and therapy of neurological disorders. LFP signals occupy a frequency band of 1 Hz to 100/200 Hz and a voltage range of 0.5–5 mV. AP signals have a frequency band of 100/200 Hz to 5 kHz with a voltage range of 50–500 μV. In addition, recording electrodes introduce background noise due to their resistance. This noise is thermal noise. Typically, noise integrated in the LFP bandwidth is smaller than 2 $\mu V_{rms}$ whereas noise integrated into the AP bandwidth is smaller than 5 $\mu V_{rms}$. On account of the dynamic range of the LFPs and APs, an ADC with an effective number of bits (ENOB) larger than 12 to 14 bits is required for recording LFPs and an ADC with an ENOB higher than 8 to 10 bits is required for recording APs.

**Figure 5:**
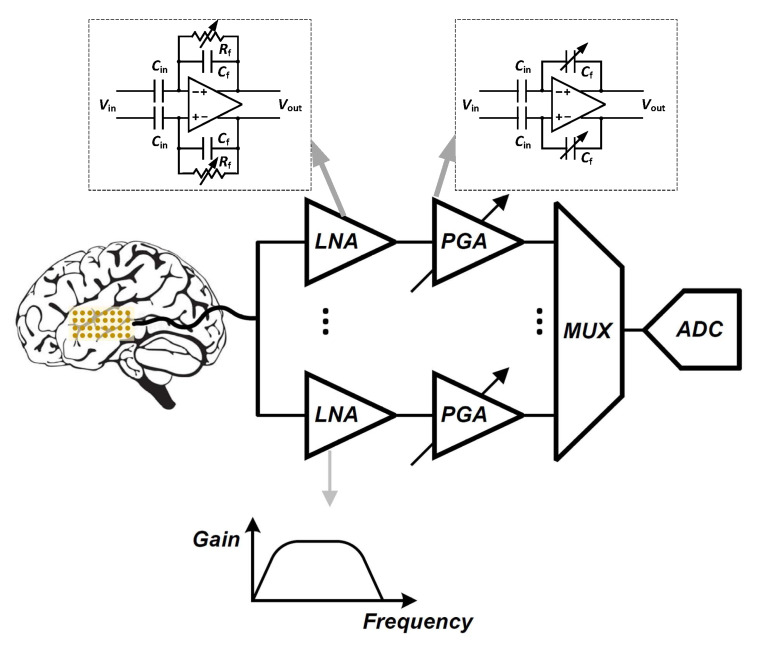
Simplified block diagram of a practical neural recording system.

Signal conditioning is the first step to accommodate the signal before digitizing it to ensure accurate reading of neural activities. As shown in Figure 5, a low-noise amplifier (LNA) is the first stage in the front-end acquisition chain, in which neural signals of small amplitudes are amplified. In this stage, the amplifier should offer a high-pass filtering behavior in order to filter out the large electrode dc offset (EDO). Classically, the gain and bandwidth of the LNA is fixed. Following the LNA, a programmable-gain and bandwidth amplifier (PGA) is used to maximally cover the input range of the ADC that follows in the signal conditioning chain. Analog-to-digital converters (ADCs) are normally used to digitize acquired signals into data prior to its further processing or transmission from the implanted device to the outside of the body. One of the most important features of a neural amplifier is its input impedance. Since most of the electrodes present a 1 kHz impedance ($Z_{1kHz}$) of less than 200 kΩ [31], the input impedance of the amplifier should be much larger than this value to accurately record the neural signals.

In a conventional sensing system, one amplifier is designed at each sensor site. To ensure matching and to reduce the level of the noise, the input stage of the amplifier consumes the largest portion of the power that is provided which allows it to satisfy matching constraints while reducing the electronic noise level. Different signal-to-noise ratio (SNR) are required to record different types of neural signals, and thus, the amplifiers are tuned for the specific targets of recording. Reducing the amplifier internal noise and its power are a trade-off that must be optimized in careful consideration of the target signals and application.

**Figure 6:**
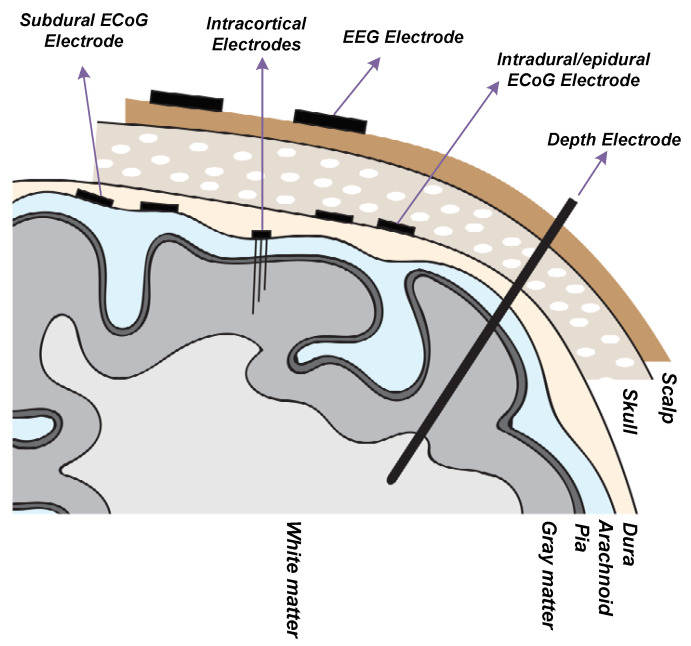
Placement of the different types of electrodes for neural recording.

Recording electrodes are required to transduce neural signals that consist of transmembrane ion exchanges into electrical signals that can be processed by microe-elctronic and information systems. The type of electrode is adapted to the target of the neural recording in the brain. Different types of neural recording electrodes are depicted in Figure 6. A brief description of different types of electrodes is provided in the following:

- Electroencephalography (EEG) Electrode ([32,33,34]): EEG electrodes are placed on the surface of the scalp. The international 10–20 system is a well-known and internationally recognized distribution of each of the EEG electrodes on the scalp. EEG recording offers several applications including brain-machine interfaces (BMI), polysomnography (PSG) for a sleep study, seizure detection, as well as other medical applications aiming at brain research. EEG recording is not an invasive method. The amplitude and bandwidth of the neural signals recorded by EEG electrodes are significantly smaller than the signals recorded by implantable electrodes due to the filtering behavior of cerebrospinal fluid (CSF), dura, skull and scalp. Furthermore, the fragile EEG signals are more exposed to different sources of artifacts including patient-related artifacts (e.g., movement, sweating, ECG, eye movements) and technical artifacts (50/60 Hz artifact, cable movements, electrode paste-related). The bandwidth of the EEG signals lies in the bandwidth of the LFP signals.
- Intracranial Electroencephalography (iEEG) [35]: Recording the neural signals inside the skull provides better signal quality in terms of signal-to-noise ratio and bandwidth. Intracranial EEG recording can be done using different types of electrodes including epidural electro-corticography (ECoG) electrodes, subdural ECoG electrodes, intracortical electrodes and depth electrodes.

**Figure 7:**
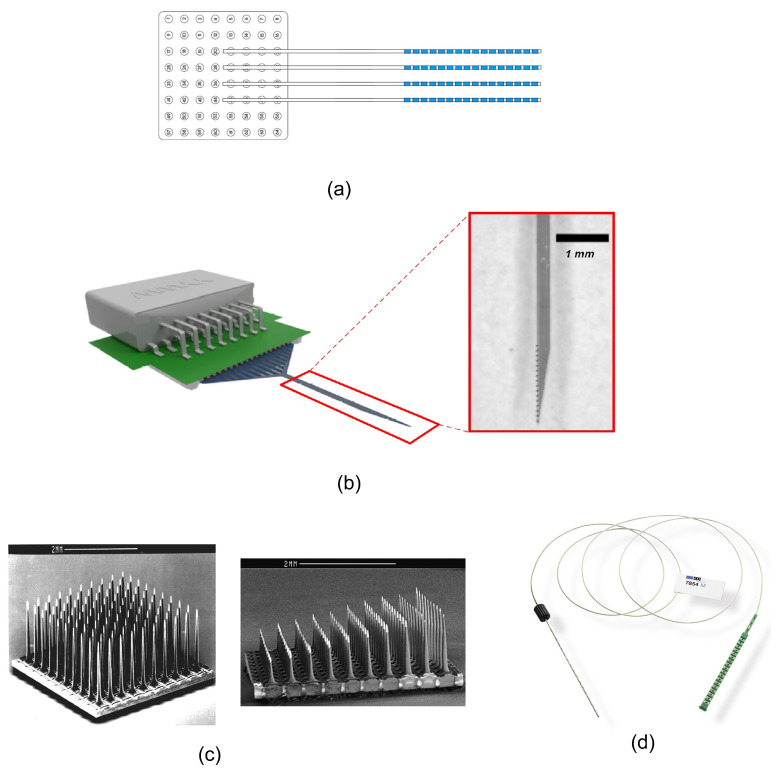
Different types of electrodes for neural recording including (**a**) grid array for intradural or subdural recording, (**b**) laminar intracortical electrodes, (**c**) intracortical micro-electrode array (Reprinted with permission of Wiley Periodicals, Inc., © 2003 The American Laryngological, Rhinological and Otological Society, Inc.), [38]) and (**d**) SEEG electrode (Courtesy DIXI medical).

Epidural ECoG electrodes [36,37]: this type of electrode is placed between the dura and skull. Hence, the dura need not be incised and opened for the placement of the electrodes on the cortex. This type of electrode is implemented in both micro-electrode and macro-electrode arrangements. Epidural electrodes are either strips of electrodes or a multicontact array as shown in Figure 7a. Epidural ECoG electrodes are suitable for both recording and stimulation.

Subdural ECoG electrodes [39]: This type of electrode is placed between the dura and the surface of the cortex. In this case, the dura must be incised for the placement of the electrodes on the cortex. This type of electrode is implemented in both micro-electrode and macro-electrode arrangements. The design of the Subdural electrodes is identical to Epidural electrodes as shown in Figure 7a. Subdural electrodes record LFP and AP signals, also depending on the size of the electrode. The main advantage of Subdural electrodes is the large coverage of the brain which enables performing a wide range of cognitive studies. Subdural ECoG electrodes are suitable for both recording and stimulation.

Intracortical electrodes [40]: intracortical electrodes are mainly micro-electrodes that are designed to record the signals from different layers of the brain. This type of electrode can perform single-unit recording, where a unique integrated circuit embeds the amplifier(s) or multi-unit recording where several integrated circuits embed multiple amplifiers, depending on the size of the electrode. In general, there are two types of intracortical electrodes including Laminar electrodes (Figure 7b) and micro-electrode arrays. These are also commonly known as neural probes or shank-based electrodes, e.g., [41]. The Utah electrode (Figure 7c) is a micro-electrode array contains up to 96 electrodes which enable high-density multi-channels recording from a large population of neurons, providing valuable data by delivering high spatial resolution within a small area of the brain.

Depth electrodes [42]: depth electrodes are placed at a precise location in the brain using a stereotactic system. Hence, this type of electrodes is also called stereoelectroencephalography (SEEG) electrodes. SEEG electrodes are suitable for recording and stimulation. Figure 7d shows a SEEG electrode manufactured by DIXI Medical [43].

### 2.3. Additional Blocks of Closed-Loop Epilepsy Control System

Neural signals that originate from several electrodes that are distributed over the surface of the cortex are digitized and can then be processed. Filtering and compressing are two classical digital processing, with which encryption has recently been complemented. In terms of the core functionality of the epilepsy control implantable system, seizures should be detected from the multi-channel recorded data. Algorithms aiming at seizure onset detection and seizure prediction should be accurate in terms of sensitivity and specificity such as to conceive durable and long-lasting IEMDs for seizure prediction and closed-loop stimulation. In addition, algorithms that are used in closed-loop stimulation systems should have a tolerable latency. An ideal seizure detector used in a closed-loop system has a sensitivity of 100% and a specificity of 100% or a false alarm rate (FAR) of zero. The importance of sensitivity is higher than specificity. Indeed, with a perfect sensitivity of 100%, a closed-loop stimulation system can detect and suppress all seizures. Improving the specificity of the seizure detector enables the closed-loop stimulation system to save power by reducing the periods of unnecessary stimulation. Furthermore, the seizures should be detected in advance or with small latency such as to be suppressed by the stimulation. Failing to satisfy the latter constraint, stimulation may not be effective for suppressing the seizures [44].

Delivering power to implanted devices is one of the major challenges in the design of IEMDs. Powering should be carried out over a wireless link, through the living tissues consisting of the scalp or skin layers. If the powering is not efficient, excessive heat due to power loss may damage the tissues around the IEMDs or may cause strange and unwanted sensations to the patients. Powering an IEMD requires applying several technologies including implantable batteries, energy harvesting or wireless power transfer. Two types of batteries are suitable for IEMDs consisting of primary batteries that are non-rechargeable, and secondary batteries that are rechargeable. Medical-grade battery equipped IEMDs are designed to include such technology. Several companies in the world provide medical batteries such as Eaglepicher [45]. Energy harvesting, which is also known as energy scavenging or ambient powering, is a process in which energy is captured from the environment and stored for further usage in small and ultra low-power systems. Energy sources may originate from external sources including solar power, thermal energy, or kinetic energy. Wireless power transfer is also a method that is suitable to power an implantable electronic system. Wireless power transfer (WPT) is also used to recharge the secondary medical-grades batteries in IEMDs.

Implantable electronic medical devices also require a wireless data transceiver. A data transceiver or a data receiver are implemented depending on the type of IEMD that is used for epilepsy control. An IEMD operating in an open-loop stimulation does not necessarily require the presence of a wireless data transmitter; however, a data receiver is needed for setting the stimulation parameters. On the other hand, an IEMD used for intracranial recording or closed-loop stimulation requires a wireless data transceiver to set the internal parameters as well as to send the recorded data to an external base station.

## 3. Commercial Systems and Products for Epilepsy Control

Commercial devices for seizure alerting and epilepsy control are reviewed in this section. These devices are considered to be partitioned into two categories, namely invasive medical devices, or implantable electronic medical devices, and non-invasive medical devices.

### 3.1. FDA Approved Implantable Electronic Medical Devices

Commercial IEMDs that are proposed in epilepsy control therapy apply electrical stimulation. These devices may operate as open-loop or closed-loop devices. In the case of open-loop stimulation, electrical stimulation is applied to the brain or group(s) of nerves without detecting any feedback from the body. On the other hand, closed-loop devices record physiological signal(s) and process them to adapt the stimuli. A closed-loop system would only stimulate the brain or a group(s) of nerves upon detection of seizure onset.

Although the quality of epilepsy detection and control of IEMDs is significantly better than it is in non-invasive medical devices, employing IEMDs presents several disadvantages, including the necessary surgery, perioperative risks, as well as side-effects such as hoarseness, throat pain, coughing, dyspnea, paresthesia, to quote the most prominent [46]. The potential of causing these symptoms enters into the decision of implanting and the selection of the device type. Governmental agencies approve devices and systems that can be implanted; these agencies are local to a country or group of countries, and include the U.S. Food and Drug Administration (FDA, USA), the Chinese National Medical Products Administration (NMPA, China; formerly the China Food and Drug Administration, CFDA), the European Economic Area CE Marking (CE marking, Europe).

#### 3.1.1. Vagus Nerve Stimulation Therapy

**Figure 8:**
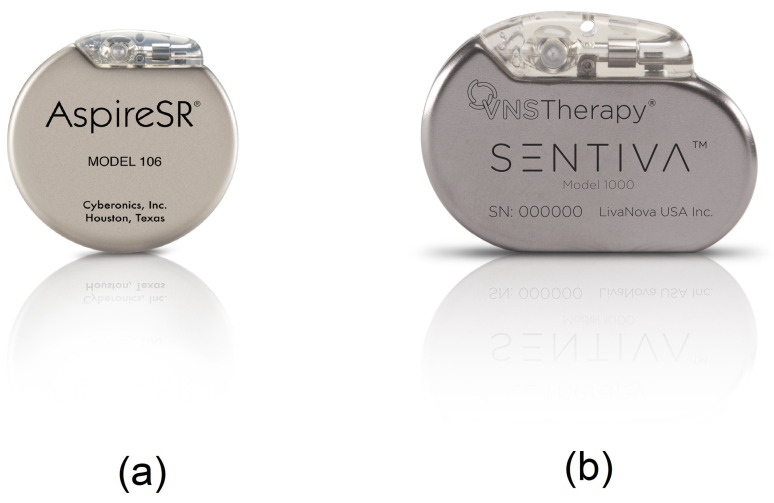
Vagus Nerve Stimulation (VNS) devices by LivaNova [47] including (**a**) AspireSR and (**b**) SenTiva, (Reprinted with permission; copyrighted material of LivaNova).

Vagus nerve stimulation (VNS) therapy was approved by the FDA in July 1997, for the treatment of epilepsy in patients who suffer from drug-resistant epilepsy. The request was presented by Cyberonics Inc. [47] which was subsequently renamed LivaNova. The VNS therapy consists of sending mild electrical pulses to the brain through the vagus nerve (generally the left vagus nerve) using an electrical pulse generator as shown in Figure 8. The stimulation parameters are set within a frequency range of 1 Hz to 30 Hz, a current range of 0 mA (no stimuli) to 3.5 mA and a pulse-width range of 130 μs to 1000 μs [48]. The vagus nerve is one of the longest nerves in the body that originates from the brain stem located on both sides of the brain. The vagus nerve is a part of the parasympathetic nervous system which is responsible for recovery and digestion, in particular. The benefits of VNS therapy may include fewer seizures, shorter seizures, better seizure recovery time, decreased seizure severity, less medication, improved mood and memory and generally improved quality of life of epileptic patients. The major adverse effects of VNS include voice alteration, coughing and shortness of breath as well as headache and neck pain. A study [49] of 60 patients with VNS therapy shows that the most common adverse effects that affect more than 20% of the patients were voice alteration (55%) and headaches (22%). In 1997, the premarket approval application (PMA) from FDA for VNS therapy (PMA number of P970003) indicated that this device is approved for use as a therapy in adults and adolescents over 12 years old. In the new version of this PMA with PMA number P970003/S207, the FDA added the patients within 4–11 years old to the VNS therapy approval. Hence, this made VNS therapy the only candidate of IEMDs that are suitable for patients less than 18 years old.

There are three types of vagus nerve stimulators for epilepsy control proposed by LivaNova including the standard model, the AspireSR and SenTiva. The standard model VNS is the earliest device designed for epilepsy therapy which only offers basic programming features. The most common stimulation pattern using this model consists of 30 s of stimulation every 5 min [50]. In the standard model, a magnet is provided to apply additional stimulation during a seizure. The new model of VNS is a closed-loop VNS based on heart rate signal acquisition and processing. This model, namely the AspireSR (Figure 8a), received the CE mark in Europe in February 2014. A study [51] of 66 seizures from 16 patients using a closed-loop VNS shows that the cardiac-based seizure detection presents more than 80% sensitivity. In addition, the severity of the seizures was significantly reduced after 3–5 days of closed-loop stimulation. In general, this study confirmed that using a cardiac-based closed-loop VNS presents an acceptable sensitivity and specificity for triggering the stimulation. The AspireSR can also apply pre-programmed stimulation throughout the day and night. The third and the newest VNS product is SenTiva shown in Figure 8b. This device offers similar characteristics as the AspireSR with some additional features. For example, SenTiva can detect if a patient is lying after a seizure, revealing a potential loss of consciousness; in addition, it can be programmed to apply a different amount of stimulation at different times of the day. The VNS is FDA-approved for MRI under specific conditions. The estimated battery life for SenTiva is 4.9–10 years [52].

#### 3.1.2. Responsive Neurostimulation

**Figure 9:**
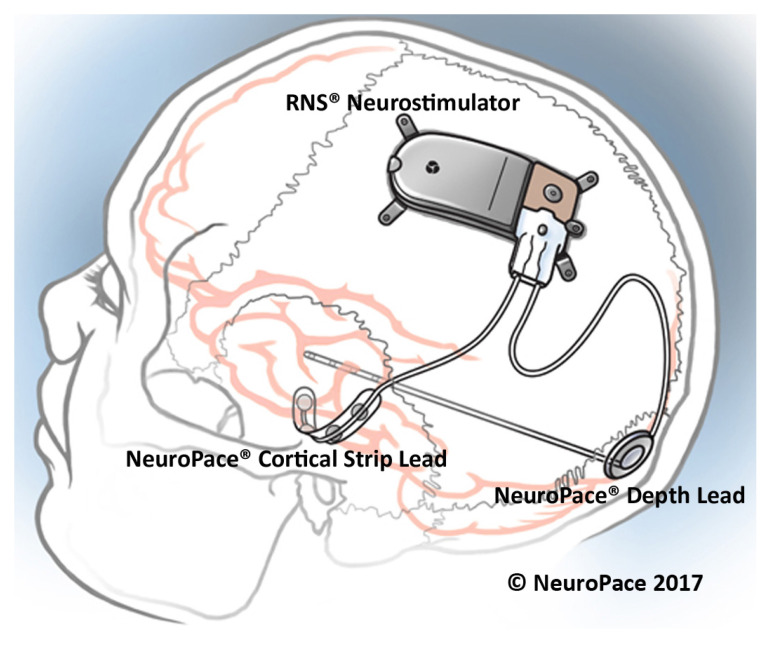
FDA approved responsive neurostimulation (RNS) system for closed-loop epilepsy detection and stimulation (courtesy of NeuroPace, Inc.) [53].

The responsive neurostimulation (RNS) therapy is a method for epilepsy control that received FDA approval in 2013. The RNS system, which is placed on the skull of the patient as shown in Figure 9, monitors the neural activity of the brain using two leads. Each lead has four electrode contacts that are used for stimulation and recording. Four amplifiers are used to the aim of recording neural signals. Each lead contains four electrodes that can be assigned to one or two out of four amplifiers [53]. The RNS system can store up to 30 min of ECoG activity [53]. The stimulation parameters are set within a frequency range of 1 Hz to 333 Hz, a current range of 1 mA to 12 mA and a pulse-width range of 40 μs to 1000 μs [54]. The current density limit of the stimulation equals 25 μC/cm${}^{2}$ in each phase, while the current density is usually much less than the limit [55]. The stimulation can be applied between any two stimulation electrodes or between the electrode and the neurostimulator case. Typical stimulation parameters include a current of 1.5–3 mA, a pulse width of 160 μs, a burst duration of 100–200 ms, and a frequency of 100–200 Hz [54].

The RNS system continuously monitors the brain signals and employs several methods to extract the appropriate feature required for seizure detection. Three algorithms are employed by the RNS system to detect seizures, namely the area, line-length and half-wave algorithms. Physicians can change the algorithms’ parameters to obtain an appropriate sensitivity, specificity and latency.

Before implanting the RNS system, the patients should undergo several tests. In order to receive the treatment from the RNS system, the patients should be older than 18 years, and suffer from disabling partial-onset seizure from no more than two foci, and they should be refractory to more than two antiepileptic medications that are properly chosen [53].

In [56], 230 patients with implanted RNS system were studied over time to measure the average decrease in seizures. This study shows that the average decrease in seizures was 44% after the first year, 53% after the second year and up to 66% after 3 to 6 years from implanting the RNS. This trend was observed among the patients who were followed over 7 years and the seizures were decreased by 72%.

#### 3.1.3. DBS

**Figure 10:**
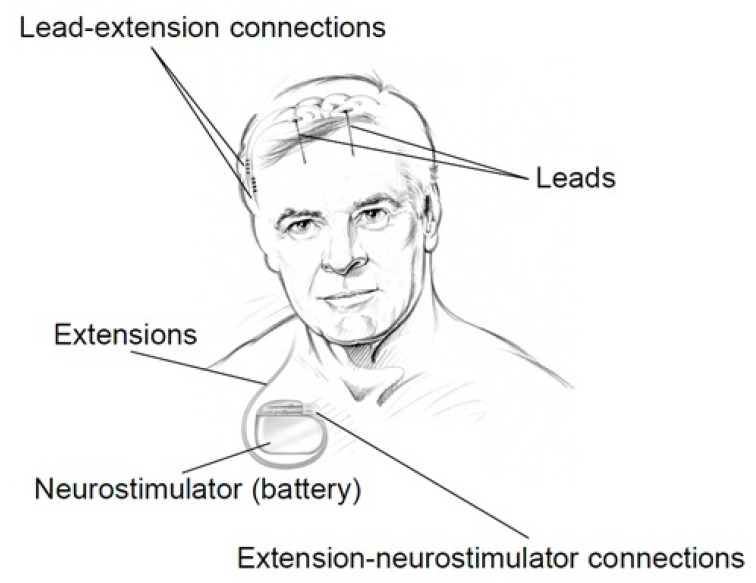
FDA approved deep-brain stimulation (DBS) system for epilepsy (Courtesy Food and Drug Administration (FDA)).

**Figure 11:**
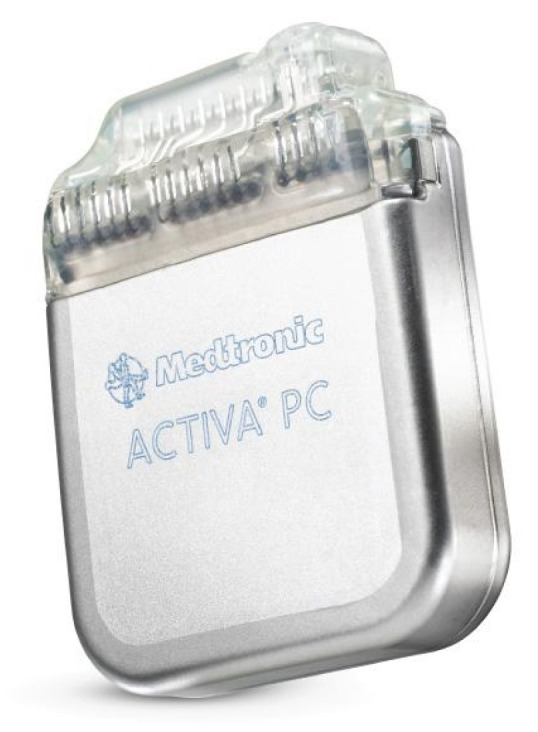
FDA approved DBS system for epilepsy [57] (Image with kind permission of Medtronic).

The Medtronic [57] Deep Brain Stimulation (DBS) system for Epilepsy which was approved by the FDA in 2018 is a device that delivers controlled electrical pulses to the brain. The system consists of a pulse generator (IPG) as shown in Figure 10 and Figure 11, that is implanted under the skin of the upper chest, and two leads implanted in the brain. The Medtronic DBS system for epilepsy helps to decrease the frequency of seizures. Unlike the RNS system, the DBS system applies an open-loop stimulation technique. The stimulation parameters are set within a frequency range of 2 Hz to 250 Hz in voltage mode, and 30 Hz to 250 Hz in current mode, a current range of 0 mA to 25.5 mA, a voltage range of 0 V to 10.5 V and a pulse-width range of 60 μs to 450 μs [58]. The Medtronic DBS system for epilepsy is used in conjunction with antiepileptic drugs in individuals 18 years of age or older, with partial onset seizures, with or without secondary generalization, who have not responded to three or more antiepileptic medications. The DBS system is used in patients who have an average of six or more seizures per month. It has not been evaluated in patients with less frequent seizures.

In [59], 157 patients with implanted DBS were studied over time to measure the average decrease in seizures. This study shows that the average decrease in seizures frequency was 56% after the second year of implantation. This study also shows that 54% of the patients had a seizure reduction of at least 50% after the second year of implantation. During this study, 14 patients were reported seizure-free for at least 6 months.

### 3.2. Commercialized Non-invasive Medical Devices

Several different commercialized non-invasive medical devices are available in the market. In the following, we mention some of these devices. In general, non-invasive medical devices belong to the two following groups, namely,

- external stimulators, and
- seizure alerting devices.

#### 3.2.1. External Stimulators

In spite of considerable advantages in terms of efficiency and patient comfort and safety, implantable electronic medical devices for epilepsy control have the disadvantage of invasiveness, which may in some cases be intolerable. In contrast to IEMDs, external stimulators are not invasive and thus, no perioperative risks are taken. External stimulators are cheap and easy to use, although they are not as comfortable as IEMDs. Two types of external stimulators have been shown to affect the seizure frequency, namely the transcutaneous Vagus Nerve Stimulation (tVNS) [60] and the External Trigeminal Nerve Stimulation (eTNS) or transcranial Trigeminal Nerve Stimulation (tTNS). In the following, each type of external stimulation is reviewed and commercialized products are presented.

**Figure 12:**
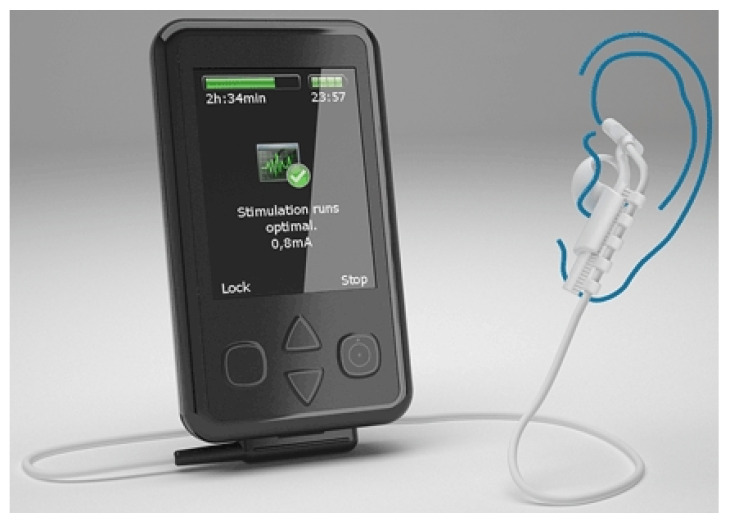
Transcutaneous Vagus Nerve Stimulation device for epilepsy control [63] (Image reprinted with kind permission of tVNS Technologies GmbH).

The effectiveness of tVNS for depressive disorders treatment is reported in [61]. One hundred and twenty cases with mild and moderate depression were studied in a double-blinded randomized clinical trial. This study shows that tVNS has the same effect as VNS in the treatment of depressive disorders. These results encouraged researchers to investigate the effects that tVNS has on epilepsy. In [62], a group of ten patients with drug-resistant epilepsy were studied. tVNS was applied while keeping the dose of the medication. Stimulation was performed using biphasic pulses of pulse-width of 300 μs with an applied average voltage of 25 V. The stimulation frequency was chosen as 10 Hz. This pilot study shows that employing tVNS could reduce the seizure frequency in half of the patients. However, this study has several drawbacks including a lack of randomized stimulation, due to a small number of patients and an inhomogeneous patient group in terms of the type of seizures (focal, generalized epilepsy). This study led to introducing the Nemos system (Figure 12), manufactured by Cerbomed [63], which was taken over by tVNS Technologies GmbH in October 2018. Nemos, which is a transcutaneous device, stimulates the auricular branch of the vagus nerve using a handheld pulse generator. The patients have to apply the stimulation four hours per day. This device received the CE marking in Europe and costs 2499.00 €. The Gammacore is another device introduced by ElectroCore Medical LLC which employs transcutaneous VNS for the treatment of epilepsy. This FDA-approved device which is mainly designed for the treatment of headaches is also suggested as a treatment for epilepsy [64].

In 2008, Dr. Christopher DeGiorgio started a project pertaining to the long-term study on the effect of external trigeminal nerve stimulation (eTNS) on epilepsy control [65]. The project started with 50 patients. Out of 50 patients, 35 patients continued the long-term study for one year. In this study, it is shown that the median seizure frequency decreases by 34.8% after one year by employing eTNS. The Monarch eTNS system is an external trigeminal nerve stimulation system proposed by NeuroSigma [66]. This device has European approval for the treatment of epilepsy in adults and children 9 years and older.

#### 3.2.2. Seizure Alerting Devices

Seizure alerting devices are designed to notify the onset of a seizure. These devices help the patients to quickly obtain help from their surroundings. Sudden unexpected death in epilepsy (SUDEP) is a fatal circumstance of epilepsy which often occurs during sleep. Seizure alerting devices help the caregivers and family by notifying the seizure onsets. Some seizure alerting devices are capable of monitoring the breathing of the patients during the sleep; upon detecting any abnormal circumstance, they notify the family or the caregiver of the patients. In addition, alerting the parents of an epileptic child is vital since a prolonged seizure can lead to brain damage, and even death. Hence, seizure alerting systems are important recent devices with a new role in epilepsy handling. Nevertheless, seizure alerting devices are by essence not useful for the patients who are alone, or patients who do not accept to be checked by others. Most of these devices detect the seizures by monitoring the repetitive movements of the patient, which may be ineffective to some types of epilepsy, e.g., tonic-clonic seizures or focal motor seizures. Hence, these devices cannot detect the seizures if the patient does not exercise large movements, e.g., during an absence seizure. Four types of seizure alerting devices are available at the time of writing:

- Watch devices
- Motion devices
- Mattress devices
- Camera devices

Most watch devices used as seizure alerting systems employ an accelerometer to detect abnormal and repetitive movements. Some of these devices have a global positioning system (GPS) device. Hence, if a seizure is detected, the location of the patient is sent by smartphone text messages or email. The Smartwatch Inspyre by Smart Monitor [67] is a smartwatch that detects the repetitive shaking motions and alerts determined contact person(s).

**Figure 13:**
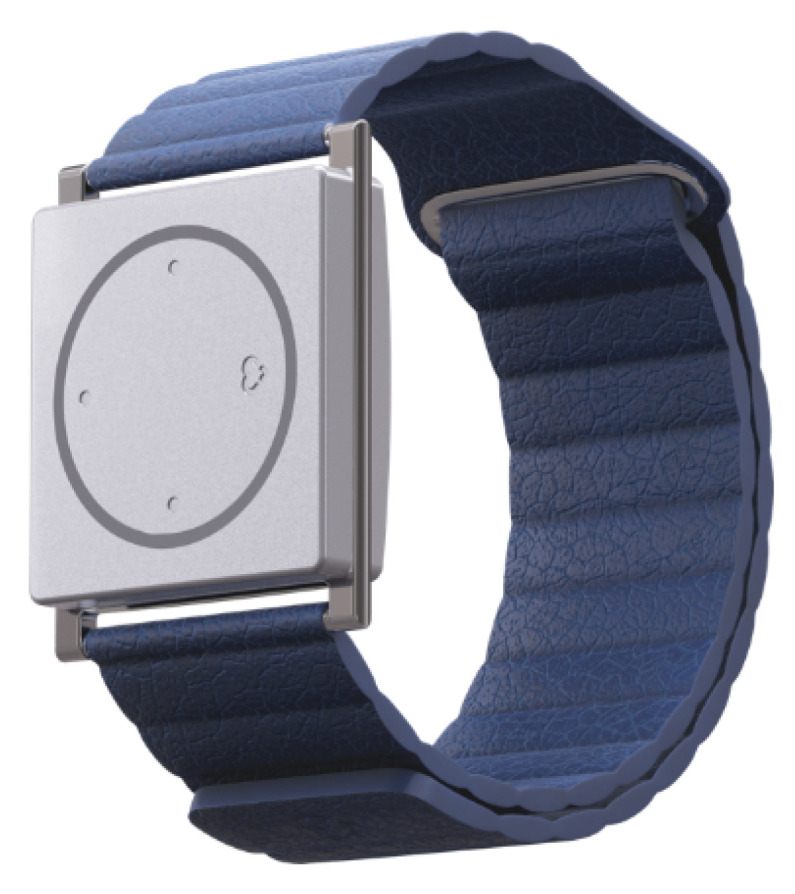
Embrace2 by Empatica Inc. [72] for seizure detection (Image reprinted with kind permission of Empatica Inc.).

The electrodermal activity (EDA) which is also known as galvanic skin response (GSR) is a biomarker that is shown to be effective in seizure detection. The EDA relates to the electrical characteristic of the skin that changes due to sweating in response to a physiological change in the body. The electrodermal activity is monitored by measuring skin conductance. For example, the conductance can be calculated by applying a low-amplitude constant voltage while measuring the current [68]. In [69], it is shown that using an EDA sensor in addition to the accelerometer used for motion/movement detection increases the quality of the seizure detection, which led to proposing new algorithms for seizure classification reported in [70,71]. This concept is used in the watch device with FDA clearance which was introduced by Empatica Inc. [72]. Embrace2 is the latest watch device for seizure alerting which monitors the EDA, temperature and also employs an accelerometer and gyroscope. This 249 $ device, which is shown in Figure 13 offers more than 48 h of battery life with fast charging capability (30 min). This device sends the data acquired by the sensors to a compatible smartphone using Bluetooth technology. The smartphone processes the data using an application and alerts the family or a caregiver if a seizure is detected. Empatica Inc. also proposes a 1690 $ device namely the E4 for scientific research. The E4 has several sensors including a photoplethysmography (PPG) sensor, an electrodermal activity sensor, an infrared thermopile for reading the skin temperature and a three-axis accelerometer. Raw data delivered by the watch can be viewed in real-time and saved for future use.

The EDA is also one of the most important biomarkers of SUDEP, as shown in [73]. It is reported that post-ictal generalized EEG suppression (PGES) appears to be a flat EEG following a seizure which is found in 100% of cases of SUDEP [73]. It is also shown that the duration of PGES correlates with the amplitude of EDA measured from the skin. This founding confirmed that seizure alerting devices such as the Embrace2 can deliver alert if there is a probability of SUDEP, which could save thousands of lives.

The first FDA cleared seizure alerting system that is not based on EEG recording is the Brain Sentinel monitoring and alerting system (known as SPEAC system) by Brain Sentinel, Inc. This device records the surface electromyography (sEMG) signals from the biceps of the patients. Hence, this device can accurately detect the seizures in case of tonic-clonic seizures. In addition to sEMG recording, the system records the audio during each event. This system helps physicians to accurately measure the seizure frequency characteristics.

Mattress seizure alerting devices are placed under a mattress where they can detect vibrations and movements. If a seizure-like movement is detected, an alarm will be triggered. The main difference between mattress devices and watch devices is that mattress devices are not wearable. The MP5-UTB is a mattress seizure-alerting device, designed by Medpage Ltd. The Emfit MM is a mattress device which is designed by Emfit Ltd. and which monitors the movements during sleep. This device was used in a study [74] including 45 patients. In this study, 78 seizures were detected using video-EEG. The Emfit MM system could detect 23 seizures out of the 78 seizures. Most importantly, this device could detect 11 out of 13 tonic-clonic seizures.

Camera devices are alternate non-wearable seizure alerting devices with which the movement of the patient is recorded and processed. The system warns the family or the caregiver of the patient if any unusual movement is detected. SAMi is a camera-based seizure alerting system by Hipass Design LLC. In this system, a remote infrared camera records the patient movements and sends the data to a smartphone for processing. This device records the audio of each event, as well.

## 4. Neural Recording Circuit Techniques

As a common block to most aforementioned systems, the front-end amplifiers in the recording chain represents one of the most challenging part. Owing to the extremely low amplitude of the recorded brain signals, low-noise electronics are required. Simultaneously, low-power design is necessary to guarantee the autonomy of the implantable and also portable systems. These constraints turn into contradictory design specifications and thus trade-offs must be accepted. While solutions have been provided, new challenges appear to emerge with the increase in the number of electrodes and recording channels. This criteria is in contrast to most other blocks of implantable systems which can be limited even to a single unit. Consequently, recording techniques show significant design challenges, which are explored in the following along with their possible solutions.

### 4.1. Low-Noise Front-End Amplifier

A neural amplifier is required next to the sensing electrode to amplify the weak neural signals and filter them. A neural amplifier should have five main features as follows:

- high-pass filtering for electrode offset rejection
- appropriate gain for conditioning the signals prior to digitization
- low input-referred noise for sensing weak neural signals
- low-power consumption (for neural amplifiers used in implantable devices)
- compact size (for neural amplifiers used in implantable devices)

In addition to the aforementioned features, a neural amplifier may provide integrated low-pass filter functionality. A low-pass filter is an important part of the analog front-end (AFE) to avoid aliasing. In general, neural amplifiers are considered into two categories, including ac-coupled amplifiers and dc-coupled amplifiers.

**Figure 14:**
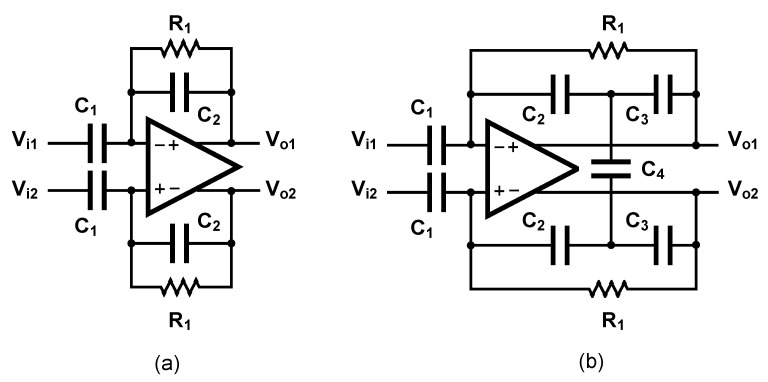
Overview of the ac-coupled capacitive-feedback structure for neural amplifiers. (**a**) Classical feedback topology and (**b**), capacitive T-feedback topology.

The ac-coupled amplifiers use a capacitor placed at the input, in the signal path to block the dc level. Several architectures of ac-coupled neural amplifiers are proposed in literature including capacitive-feedback network neural amplifiers, open-loop network neural amplifiers and chopper amplifiers. Figure 14 shows two common architectures for designing ac-coupled capacitive-feedback neural amplifiers.

**Figure 15:**
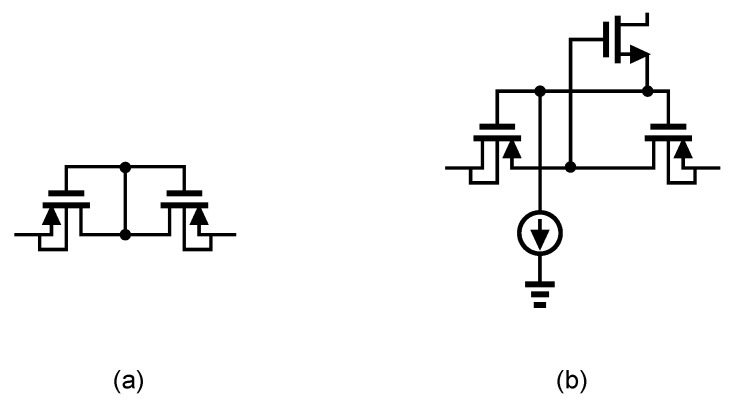
Pseudo-resistor architectures. (**a**) Fixed-value and (**b**) tunable pseudo-resistor.

Figure 14a presents the general architecture of ac-coupled capacitive-feedback neural amplifiers. In this structure, $C_{1}$ is used to block the dc level that is created at the electrode-electrolyte interface. The value of $C_{1}$ is typically selected smaller than 20 pF since this capacitor affects the input impedance of the amplifier. The mid-band gain of this amplifier is defined as $C_{1}/C_{2}$. There is a high-pass corner in the frequency response of this amplifier which is determined by $C_{2}$ and $R_{1}$. For recording low-frequency neural signals, the high-pass corner frequency of the amplifier should be at few MHz to few Hz. Hence, a very large resistance is required to achieve such a corner frequency. In neural amplifiers used in implantable devices, these resistors are implemented as pseudo-resistors to avoid bulky physical resistors. A pseudo-resistor is implemented as a highly-resistive triode-biased MOS transistor as shown in Figure 15a [75]. This type of resistors is highly susceptible to process, voltage and temperature (PVT) variations. In addition, pseudo-resistors are highly non-linear resistors and placing such a resistor between the input and output of an operational transconductance amplifier (OTA) may yield a voltage-dependent resistor. If the voltage swing across this resistor is large, then its value may change significantly with respect to the voltage across it, and thus create a voltage-dependent high-pass pole for the neural amplifier. In addition, the structure shown in Figure 15a is not tunable and only creates one value of resistance. In order to design a neural amplifier with an adjustable high-pass pole, a tunable pseudo-resistor is required as shown in Figure 15b. The voltages across the Gate-Source of the PMOS transistors are set using an NMOS and a current source, such that the equivalent resistance changes [76].

For achieving a high-gain amplification in the structure shown in Figure 14a, $C_{2}$ is typically selected very small. Lowering the value of $C_{2}$ affects the common-mode rejection ratio (CMRR) of the amplifier as well as the gain precision. Figure 14b shows an architecture that is similar to Figure 14a, yet with a minor topological difference. In Figure 14b the capacitor -feedback network is implemented using a capacitive-T topology. This topology allows the implementation of a low-value of the feedback capacitor using higher values of capacitors [77]. This technique improves the matching between feedback capacitors, thus improves the CMRR of the amplifier. The equivalent feedback capacitor in this architecture is calculated as:

(1) $$C_{efb}=\frac{C_{2}C_{3}}{2C_{4}+C_{2}+C_{3}}.$$

**Figure 16:**
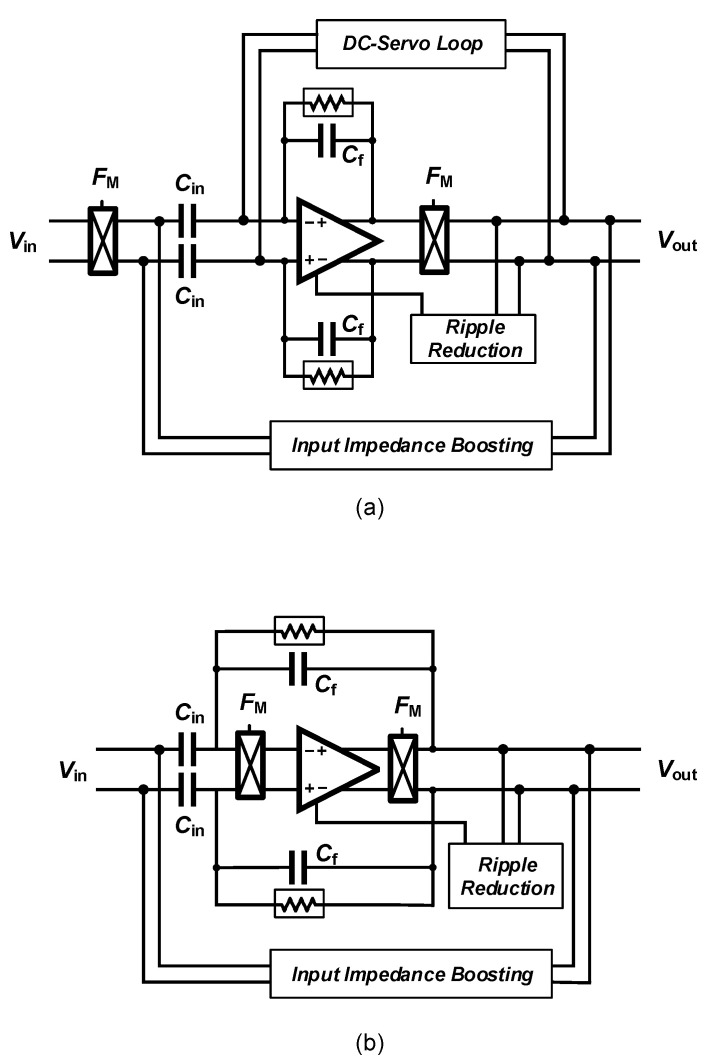
The ac-coupled chopper amplifier architectures. (**a**) Classical topology with servo loop and (**b**) alternated topology without servo loop.

Amplifying LFP signals using a neural amplifier creates significant issues related to Flicker noise, also named 1/f noise that lies in the same frequency range. In order to deal with this issue, chopper amplifiers are widely used for sensing LFP signals. However, ac-coupled chopper amplifiers carry over new issues which require new circuit and systems solving techniques. The classical architecture of a chopper amplifier is shown in Figure 16a. Chopper switches are placed before the ac-coupling capacitors. Hence, the dc value of the signal is modulated to a higher frequency and the ac-coupling capacitors can not filter it out. In order to filter the dc component of the input signal, a dc servo-loop is used in this circuit. Furthermore, chopper switches used in conjunction with ac-coupling capacitors reduce the input impedance of the amplifier. An impedance-boosting circuit must be used in the architecture of the chopper amplifier. Another issue of this circuit relates to the ripples introduced into the signal path by the chopper switches. This issue is addressed using a ripple-reduction circuit in the architecture of chopper amplifiers.

An alternate architecture employing a chopper amplifier is shown in Figure 16b. The chopper switches are placed after the ac-coupling capacitors in the signal path. Hence, this architecture does not require a dc-servo loop to block the dc component of the input signal. However, placing the chopper switches after the ac-coupling capacitors results into shaping the OTA thermal noise with 1/f characteristic when referred to the input of the neural amplifier [78]. Hence, this architecture requires a very high value of $C_{1}$ (in the order of 300–500 pF) to degrade this effect. In [79], ac-coupling capacitors are implemented using off-chip capacitors.

**Figure 17:**
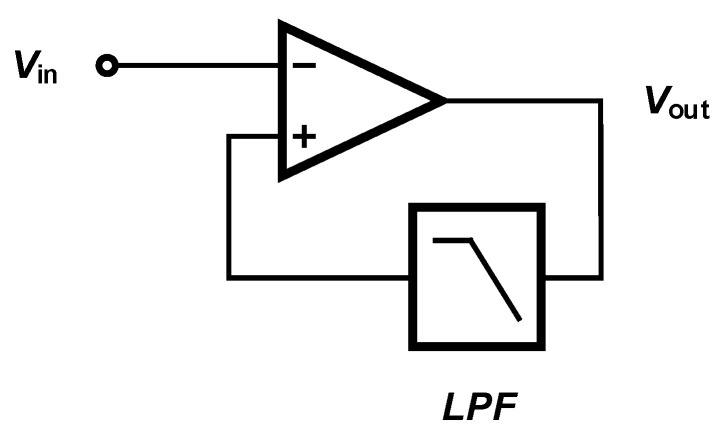
General architecture of dc-coupled amplifiers.

In contrast to ac-coupled amplifiers, dc-coupled amplifiers use a low-pass filter to block the dc component of the input signal, as shown in Figure 17. Several methods are introduced in the literature to implement a dc-coupled amplifier. The low-pass filter used in the structure of a dc-coupled amplifier can be implemented in analog [80], digital [81] or a combination of the domains [82].

#### Amplifier Sharing Methods

**Figure 18:**
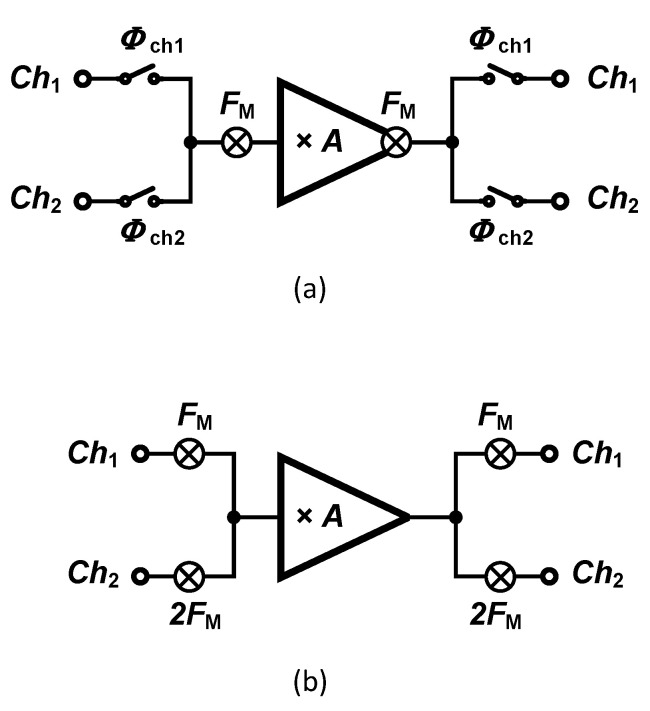
Amplifier sharing techniques: (**a**) time-division multiplexing (TDM) technique proposed in [84] and (**b**) frequency-division multiplexing (FDM) technique proposed in [85].

A technology trend is currently observed towards increasing the number of recording sites, which in turn creates severe constraints to the amplifier-related microelectronics. Hence, alternate solutions must be explored to satisfy the constraints in dense multichannel designs in terms of silicon area and power consumption. Techniques supporting sharing one amplifier among multiple channels become attractive as the number of recording channels increases. The sharing technique can be either applied at system-level or circuit-level. In circuit-level techniques, the amplifier is not totally shared and the current that is provided to an input differential pair circuit is reused by the other channels’ input pairs. This method, which is called orthogonal current reuse technique, is introduced in [83]. System-level techniques are widely presented in the literature. Two different methods aiming at sharing a single amplifier are introduced in literature, namely the time-division multiplexing (TDM) [84] and frequency-division multiplexing (FDM) [85] techniques which are shown in Figure 18a,b, respectively. Several critical design challenges yield from these sharing techniques, such as crosstalk, settling, accuracy, and filtering. If the amplifier used in TDM is not fast enough, it may introduce large crosstalk between channels. the timing of all signals is very critical in TDM. As discussed in [86], in addition to the crosstalk, timing issues may cause settling errors during the analog-to-digital conversion, in turn causing noise aliasing degrading the noise performance of the circuit.

**Figure 19:**
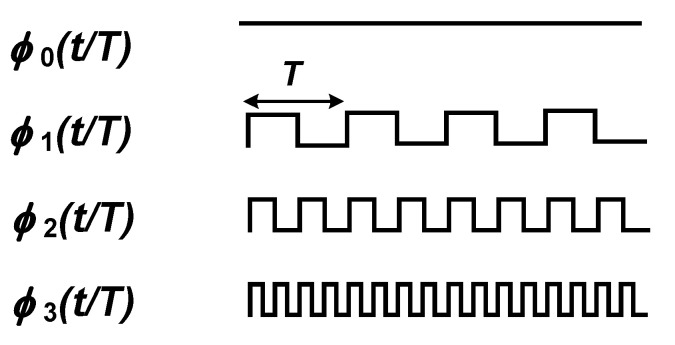
First four Rademacher functions.

The first chopper amplifier based on FDM was proposed in [85]. As shown in Figure 18b, inputs are modulated using different chopping frequencies that are orthogonal. In order to guarantee the orthogonality between chopping frequencies, $2^{n}\times F_{M}$ is proposed, in which n=0,…,N−1 and *N* is the number of channels. Rademacher functions are actually applied, which are sequences of orthogonal functions that have two values of ±1 and are defined by the conditions expressed in Equation (2).

(2) $$\begin{array}{c} \begin{array}{c} \phi_{0}\left(\frac{t}{T}\right)=1\;\;\;\;\;\;\;\;\;\;\;\;\;\;\;\;\;\;\;\;\;\;\;\;\;\;\;\;\;\;\;\left(0\leq \frac{t}{T}<\frac{1}{2}\right)\\ \phi_{0}\left(\frac{t}{T}\right)=-1\;\;\;\;\;\;\;\;\;\;\;\;\;\;\;\;\;\;\;\;\;\;\;\;\;\;\;\;\left(\frac{1}{2}\leq \frac{t}{T}<1\right)\;\;\\ \phi_{0}\left(\frac{t}{T}+1\right)=\phi_{0}\left(\frac{t}{T}\right)\\ \phi_{n}\left(\frac{t}{T}\right)=\phi_{0}\left(\frac{2^{n}t}{T}\right)\;\;\;\;\;\;\;\;\;\;\;\;\;\;\;\;\;\;\;n=1,2,\ldots \end{array} \end{array}$$

in which $\phi_{n}$ is called the *n*th Rademacher’s function and *T* is the period of the functions. The waveform of the first four Rademacher’s functions are shown in Figure 19. Except $\phi_{0}$, Rademacher’s functions can be used as the modulating signal in a chopper amplifier based on FDM. Nevertheless, the technique proposed in [85] presents some major drawbacks which are discussed in the following.

A significant issue regarding the usage of different chopping frequency for different channels relates to the mismatch between the input impedance of the channels. An additional issue of the technique relates to the need for a low-pass filter after the demodulation of each channel. Since the chopper is located after the opamp, high-frequency signals that remain in the demodulated signal (Flicker noise and offset) are not filtered out. Therefore, an additional low-pass filter is required to clean the output signal from high-frequency contents. A third issue of the technique relates to the gain mismatch between channels which in turn relates to the bandwidth of the opamp used in the chopper amplifier.

**Figure 20:**
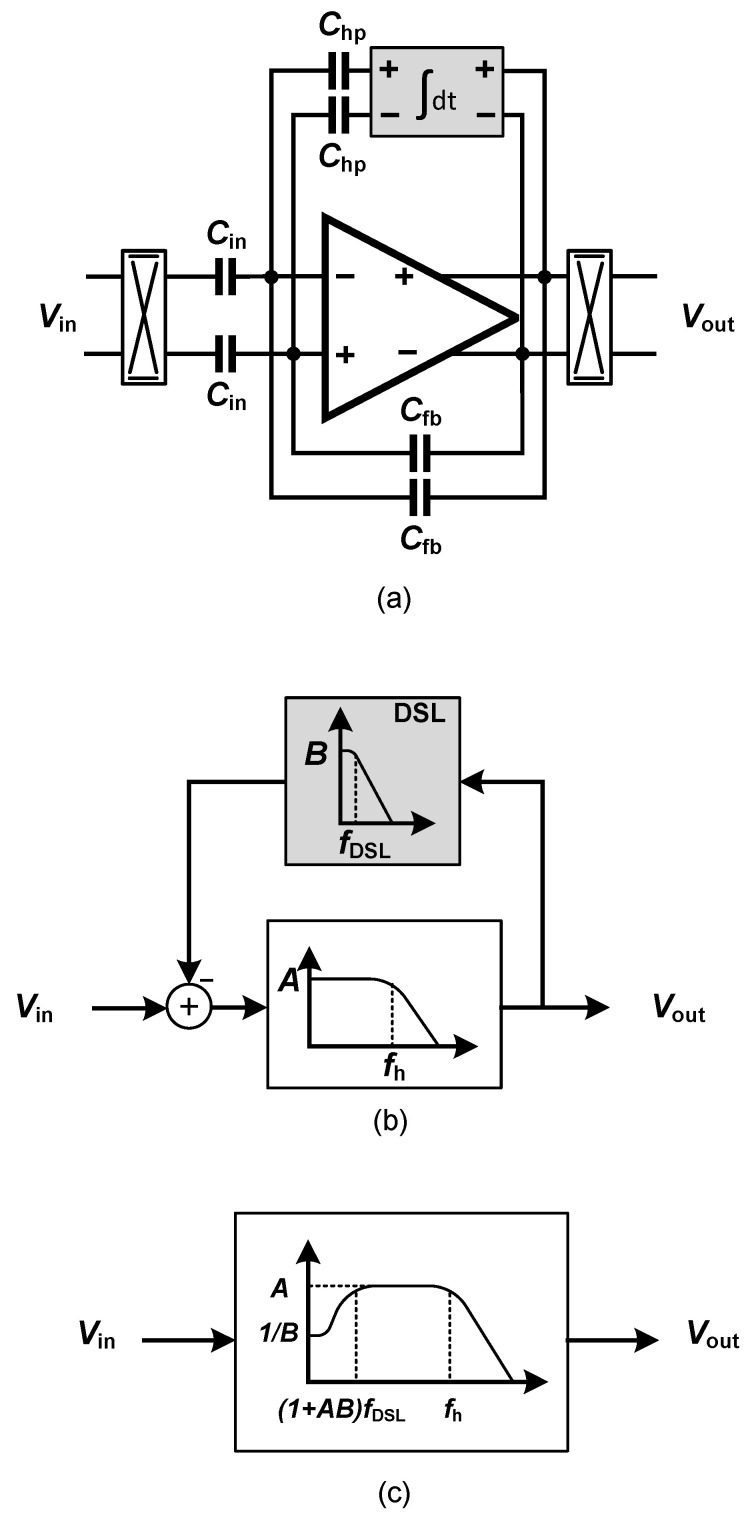
Effect of the dc servo loop on the overall frequency response of the chopper amplifier. (**a**) Topology including a dc-servo loop. (**b**) Block-level frequency responses of the topology including a servo loop. (**c**) Overall frequency response.

The usage of a two-channel chopper amplifier proposed in [85] as a neural amplifier presents a significant drawback. The lack of a dc servo-loop indeed represents a severe issue of two-channel chopper neural amplifiers. A large electrode dc offset can easily saturate the amplifier if it is not filtered. In chopper amplifiers, the dc servos loop (DSL) is employed to implement a high-pass corner in the frequency response of the chopper amplifier, as shown in Figure 20a. Figure 20b,c illustrate the effect of the DSL on the overall frequency response of the amplifier, using control theory. The DSL introduces a high-pass corner at a frequency of $\left(1+AB\right)f_{DSL}$, in which $f_{DSL}$ is the bandwidth of the integrator used in the DSL. At the circuit level, (Figure 20a), the high-pass corner is calculated using Equation (3).

(3) $$f_{hp}=\frac{C_{hp}}{C_{fb}}f_{0DSL},$$

in which $f_{0DSL}$ is the unity-gain frequency of the integrator in the DSL.

**Figure 21:**
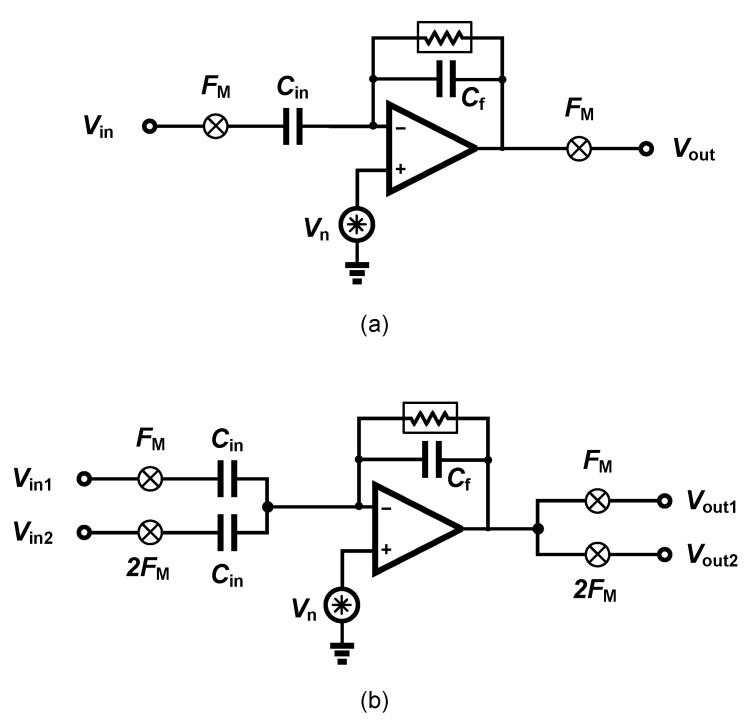
Noise analysis model of (**a**) a single-channel chopper amplifier and (**b**) a two-channel chopper amplifier.

The noise performance of a two-channel orthogonal chopper amplifier is also degraded with respect to the single-channel amplifier. In a single-channel amplifier as shown in Figure 21a, the signal gain from input to the output is equal to −$C_{in}/C_{fb}$ and the noise gain from the amplifier input to the output ($V_{out}/V_{n}$) is equal to 1 + $C_{in}/C_{fb}$. In a two-channel chopper amplifier as shown in Figure 21b, the signal gain from the input to the output is equal to −$C_{in}/C_{fb}$; however, the noise gain from the amplifier input to the output ($V_{out}/V_{n}$) is equal to 1 + 2$C_{in}/C_{fb}$. Therefore, the input-referred amplifier noise of a two-channel chopper amplifier is approximately two times higher than single-channel chopper amplifier.

### 4.2. Data Compression

Data compression is a well-known method to reduce the power consumption of wireless transmitters in implantable electronic medical devices. Using data compression also reduces the power consumption of feature extractors as shown in [87]. Compressive sensing (CS) is a compression scheme presenting relevant characteristics for multi-channel neural recording. This emerging compression method has lower complexity in comparison to established compressing methods. Compressive sensing reduces the number of measurements for a high-dimensional signal with respect to the number of measurements dictated by the Nyquist sampling theorem. Compressive sensing can be applied in three different domains, including analog, digital and multichannel. Analog compressive sensing (ACS) is a method applied to reduce the data rate and digitization power. However, due to the multi-path nature of this technique, a large on-chip silicon area is required. Digital compressive sensing (DCS) is shown to be a superior method over ACS in terms of power consumption [88]. However, this technique requires several accumulation blocks for each channel, which is not tractable in a multi-channel system with a limited silicon area.

**Figure 22:**
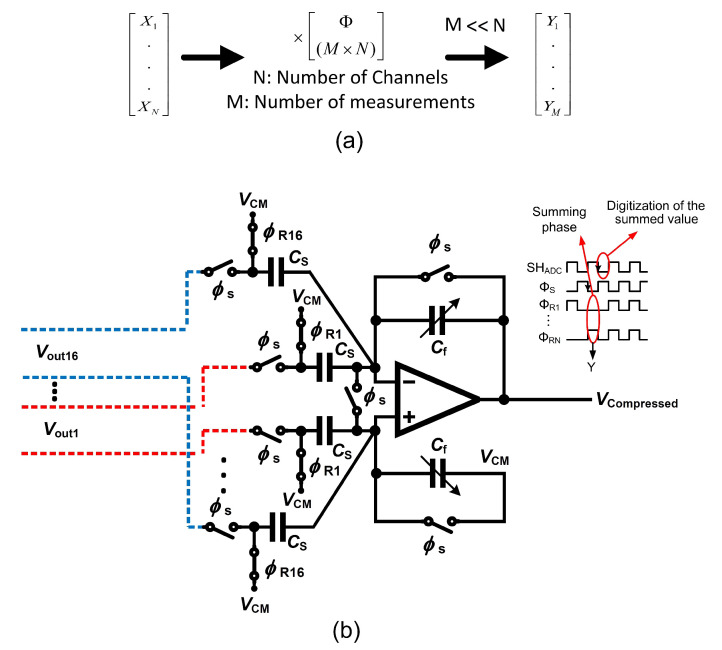
Multichannel compressive sensing (MCS). (**a**) Overview of the MCS concept. (**b**) Structure of the conventional multi-input single-output compressive sensing (MISOCS) block with 16 inputs.

The multi-channel compressive sensing (MCS) technique has been developed as a hardware-suitable compressive sensing that allows a straightforward circuit design as well as an efficient power × area performance [87]. Figure 22a depicts the simplified concept of the MCS operating over *N* channels. Φ is a measurement matrix that consists of a random sequence of ones and zeros, striclty. Φ is used to project a matrix that stores data from *N* channel into the compressed domain. The result of the operation is a vector comprising data that is the compressed image of the data recorded from *N* channels.

Circuit area and power dissipation can be optimized by applying the MCS at the sensing node. The complexity is deferred to the receiver that applies the data reconstruction algorithm from compressed data. The complex data reconstruction operation is performed in the digital domain and involves a significant latency which would slow down any seizure pattern detection or further feature extraction processes. Feature extraction from spatially filtered data based on the MCS is presented in [87] to the aim of detecting seizures. A multi-input single-output compressive sensing (MISOCS) block carries out the projection of data originating from *N* channels into the compressed domain. The MISOCS block processes the input channels in parallel owing to its implementation as a summing amplifier, which is detailed in the following.

Figure 22b presents the system architecture of a 16-channel (N=16) conventional MISOCS block and the signals that control the corresponding circuit. A compression ratio of 16 (CR=16) is achieved by the system [87]. The 16 inputs to the MISOCS block originate from the front-end neural amplifiers differential outputs. The control signals of the MISOCS block consist of a sampling signal $\phi_{s}$ as well as a measurement matrix $\Phi \in R{}^{1\times 16}$. Sixteen random sampling signals $\phi_{R1},\ldots ,\phi_{R16}$ form the measurement matrix. The conventional MISOCS block operates in two phases. Signal $\phi_{s}$ controls the sampling phase during which the output of the individual channels are sampled. $\phi_{s}$ at the LOW level marks the summation phase. The value presented on the channels that correspond to a random sampling value equal to one in the measurement matrix are accumulated at the output node of the summing amplifier. This conventional architecture of the MISOCS block is straightforward but suffers from circuit-level intricacies.

First, the random nature of the measurement matrix yields a random number of ones in the random sampling signals [87]. The offset of the summing amplifier depends on the number of ones, thus causing an error. In practical terms, a random signal which has a value between zero and $V_{os}\left(1+\frac{KC{}_{in}}{C_{f}}\right)$ is summed up with the compressed signal, at the output of the summing amplifier. The error is random in nature and derives from the implementation of the method at the circuit level. The random error causes a degradation of the seizure detection efficiency which is due to a degradation of the quality of the signal reconstruction.

In addition, careful consideration must be devoted to controlling the dynamic range of the compressed signal, as it is generated at the output node of the summing amplifier. The dynamic range of the compressed signal at the output of the MISOCS block is calculated in the following, considering the two extreme cases. The first extreme case consists of a full correlation of input signals (C=1) while the random sampling signals are all at one. This situation yields an output corresponding to a linear sum of all inputs and thus the highest level of the compressed signal. In the highest level worst case, all the *N* random sampling signals are at one; thus, the output consists of a linear sum of the input resulting in a highest value of the compressed signal that is *N* times larger than a single channel. The second extreme case consists of a single random sampling signal at one, while all others are at zero. This situation yields the lowest level of the compressed signal which is meaningful, and corresponds to the RMS noise of a single channel analog front-end (AFE).

The dynamic range of the compressed signal can be expressed from the highest and the lowest meaningful levels that are acceptable at the output node of the amplifier. Consequently, the dynamic range of the compressed signal in an N-channel conventional MCS system is *N* times larger than a single AFE channel. The dynamic range of the compressed signal is thus used in the calculation of the resolution of the ADC that follows the MISOCS block, as expressed in Equation (4).

(4) $$B_{ADC}\approx B_{Channel}+\log_{2}N,$$

where $B_{Channel}$ is the required equivalent resolution of the AFEs.

For example, a 16-channel acquisition system with an equivalent bit-resolution of each channel assumed to be equal to 8-bit yields the necessity of a 12-bit ADC to accurately reconstruct the compressed signal.

**Figure 23:**
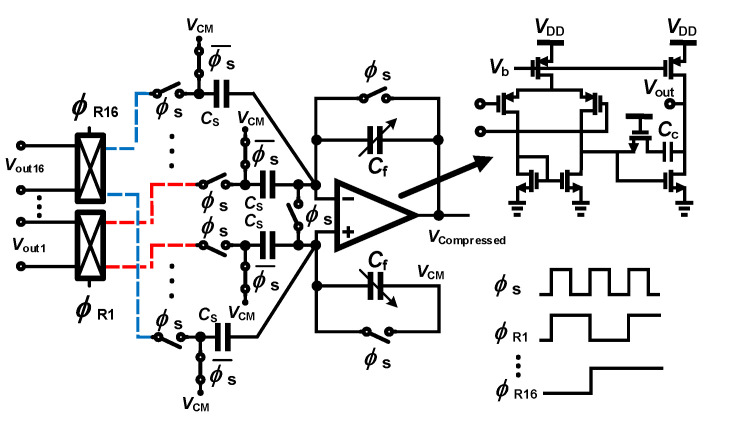
Schematic of the proposed multiple-input single-output compressive sensing block, mMISOCS.

The mMISOCS block is presented in the following [89] as a modified MISOCS block aiming at overcoming the issues of the conventional architecture that were described above. The mMISOCS architecutre is shown in Figure 23. The circuit topology includes a butterfly switch which is used to invert the output of a channel $V_{out,i}$ when the corresponding random sampling value is at zero; the result is then summed up with other channels output during the summing phase. Hence, in contrast to the conventional MISOCS block, the mMISOCS architecutre generates compressed data that is composed of data originating from all channels, i.e., not a random number of channels with coefficient at one. As a result, the noise level of the compressed data is fixed at $\sqrt{N}$ times the RMS noise of a single AFE. Consequently, the dynamic range of the compressed signal is also fixed at $\sqrt{N}$ times larger than the dynamic range of a single AFE. The ADC resolution is thus expressed in Equation (5).

(5) $$B_{ADC}\approx B_{Channel}+\log_{2}\sqrt{N}.$$

As an additional benefit of the mMISOCS topology, the offset signal that appears in the compressed signal for any combination of the values of the sampling signals is fixed because all input signals participate in the output node summation in a positive or negative value. The offset that appears at the output of the summing stage is expressed in Equation (6)

(6) $$V_{os,err}=V_{os}\left(1+16\frac{C{}_{in}}{C_{f}}\right).$$

Consequently, the error is constant and can thus be canceled by processing in the digital domain.

### 4.3. Feature Extraction

Feature extraction has an important role in the quality of the seizure detection [90]. Several classical features are introduced, which must be implemented in the digital domain, in an implantable epilepsy control device.

Considering a one-dimensional signal representing the electrical activity recorded from the brain, features can be extracted in the time and spectral domains.

- Time-domain feature extraction. The raw signal originating from the signal conditioning chain to the ADC is in turn processed. Usually, the algorithms process the data delivered as successive windows comprising a fixed number of samples. The processed feature score is compared to a threshold yielding a decision.
- Frequency-domain features. Spectral-based feature extractors operate in the digital domain. A fast-Fourier transform is typically applied to the input signal originating from an ADC, and prior to extracting features in the frequency domain. Bandwidths of interests are determined, and the energy is computed within a selected frequency range, e.g., [91]. The process is repeated in time (or time-window) yielding a decision.

Features are typically patient-specific, and thus parameters must be tuned such as to improve the accuracy of the decision. Recently, artificial intelligence and machine learning techniques also including deep-learning methods have been applied to support the feature extraction and classification, Refs. [92,93,94]. Moreover, the success of advanced AI and deep-learning algorithms in epilepsy detection has opened the way to epilepsy prediction, where interictal signals that are observed between seizures are studied with the aim of extracting reliable markers of a future seizure [95].

The hardware resources that are involved in frequency-domain feature extractors as well as deep-learning and machine-learning methods are important. The nature of the algorithms dictates a large number of memory accesses, multiplications, as well as non-linear operations processed using lookup tables. All of the aforementioned operations are time- and energy-consuming. In addition, the algorithms are developed such as to accurately operate over 32-bit number formats, while reducing the bit representation decreases the reliability. Hence, these techniques are considered not suitable for implantable devices, as excessive consumers of hardware resources and power. Recently, complex deep-learning and machine learning algorithms find adaptations to portable hardware that are dictated by new requirements of internet-of-things. Nevertheless, implantable devices require low-power blocks and feature extraction methods which are best found as the time-domain features presented in the following [96].

- Energy: the energy feature is a popular feature. The average energy of *d* samples is calculated as expressed in Equation (7).
  (7) $$Average\;\;Energy=\frac{1}{d}\sum_{d}x^{2}\left[n\right].$$
  A multiple and accumulate block is used to process the data that streams-into. The inputs of the multiplier are identical, yielding the $x^{2}$ operation.
- Accumulated energy: the accumulated energy extractor applies the energy criteria over several time-windows.
- Variance and Hjorth variance The variance criteria has extensively been applied in EEG studies. The variance is processed over a window of *d* samples, and then averaged. The intuitive formulation that directs the hardware implementation is expressed in Equation (8).
  (8) $$Variance=\frac{1}{d}\sum_{d}x^{2}\left[n\right]-\mu^{2}.$$
  The hardware is more complex than the hardware required in the energy extractor, and consists of multipliers and accumulators, subtractors and temporary storage registers.
- Line-length or Coastline: the line-length is a measure of the absolute value of the length between two consecutive data points. Line-length is a feature that increases with low-amplitude while high-frequency signals are presented, as well high-amplitude while low-frequency signals are presented. The line-length feature for *d* samples is calculated as expressed in Equation (9).
  (9) $$Line-length=\frac{1}{d}\sum_{d}\left|x[n]-x[n-1]\right|$$
  The hardware is relatively straightforward and consists of multipliers and accumulators, temporary storage registers as well as multiplexers.
- Area: area is a popular feature for seizure detection. The simplicity of the algorithm enables a low-cost and accurate seizure detection. Area is one of the features used in RNS (Section 3.1.2. The area feature for *d* samples of the signal is calculated as expressed in Equation (10).
  (10) $$\begin{array}{c} Area=\frac{1}{d}\sum_{d}|x^{}\left[n\right]| \end{array}$$
- Non-linear autocorrelation: non-linear autocorrelation feature extraction is based on detecting and accumulating the minimum of the maximum of the samples in three consecutive windows, also detecting and accumulating the maximum of the minima of the samples in three consecutive windows, and finally subtracting the latter from the former result, as expressed in Equation (11).
  (11) $$\begin{array}{c} HV=min(max\left(X_{win_{i}}\right),max\left(X_{win_{i+1}}\right),max\left(X_{win_{i+2}}\right))\\ LV=max(min\left(X_{win_{i}}\right),max\left(X_{win_{i+1}}\right),max\left(X_{win_{i+2}}\right))\\ Autoc=\sum_{3}HV-LV. \end{array}$$
  The hardware requires many resources including a multiplier-accumulators, subtractors, storage resisters as well as several comparators.

## 5. Discussion

Several challenges must be solved in open and closed-loop IEMDs, from the point of view of microelectronics engineering. Limitations are imposed from the technical and engineering perspectives, and are combined with limitations imposed by medical regulations, methods and ethical concerns. Some of the relevant open issues are summarized in the following.

- Size: the most important challenge of an implantable system is the size. Any implantable medical device (IEMD) is composed of several electrical modules. Some of these modules consist of off-chip components such as wireless powering modules or the wireless data transmitter. The specification of these modules should be defined in a way that the IEMD system presents an acceptable size. The IEMD weight is a related parameter. Increasing the size and the weight of IEMDs also increases the complexity of the surgery. Hence, for the convenience of the patients, IEMDs should have a minimum number of off-chip components in order to present a minimum size and weight.
  A solution for decreasing the size of an implant is to integrate the active circuits as close as possible to the electrode. One method to realize this solution consists of fabricating a silicon-based electrode which allows the active circuits to be implemented on the same silicon or by attaching the active circuitry to the silicon-based electrode using post-CMOS processes.
- Power consumption and temperature elevation: a limitation for temperature raise is imposed by medical regulations for IEMDs. IEMDs temperature should not exceed predefined limits. Generally, the temperature of the outer surface of an implanted device must be limited to 2 °C above body temperature [97]. However, this limit is reported to be 1 °C above body temperature in IEEE standards [98], specially in cortical implants [99]. A device that exceeds this limit should be turned off immediately. Hence, temperature sensors should be considered in the design of IEMDs and stimulators to enable temperature management capabilities of the systems [100].
- Battery powering and rechargeability: IEMDs should offer freedom to patients to proceed in their life with regular activities. This autonomy cannot be provided without using an implanted battery. Moreover, to increase the lifetime of IEMDs, the battery should be rechargeable. Therefore, patients undergo less surgery for the placement and/or removal of the IEMDs. However, the main challenge in the design of rechargeable IEMDs consists of wirelessly and efficiently recharging the implanted battery. Efficiency in the wireless battery charging is very important since this procedure may generate heat and cause skin burning or unpleasant feeling during the battery charging process.
- Biocompatibility: the package and enclosure of IEMDs must be bio-compatible. A biocompatible package serves as a barrier between the electronics and other chemical materials to which a biological system may adversely react. The host response to an implanted IEMD (resulting from tissue trauma during the implantation of an IEMD and the presence of the device in the body [101]) depends on the type of material that is used for the packaging and the enclosure of the IEMD. The importance of the biocompatibility lies in the fact that the systemic toxicity impairs the entire biological system such as the nervous or the immune system [101]. In addition, the reason for a systemic reaction due to the biocompatibility cannot be traced back to its origin since it generally takes place at a location far from the point of contact of an IEMD. Due to all aforementioned issues, biocompatibility has become the most important part of the U.S. FDA approval procedure, even for Class I devices (lowest risk). Furthermore, biocompatibility is the major part of acquiring an ISO (International Organization for Standardization) standard such as ISO 10993.
- Data storage: in order to increase the accuracy of the seizure detection as well as to provide freedom and autonomy to the patient during the recording period, the implant should store data over a few hours. This feature is important since patients should not have to wear any bulky holder of an external unit (helmet, belt) during some specific activities or during sleeping. Hence, the system should save the recorded data on an implanted memory. If the IEMD is powered by a rechargeable battery, the IEMD should save the recorded data on a non-volatile memory since the IEMD may be turned off by the under-voltage lockout detection circuit.

While many of the aforementioned challenges find solutions at circuit and system levels, we observe a trend aiming at efficiently solving these issues considering the circuit and the system as a whole, i.e., some part of the trade-offs is solved exploiting specific circuit characteristics, while other parts of the trade-offs find solutions exploiting algorithms or taking benefits of system-level improvements.

## 6. Conclusions

This review paper presents and discusses the major blocks of the signal recording and seizure detection of an implantable system aiming at epilepsy control. While classical techniques pertaining to analog and digital circuit design are used, the paper focuses on specificities related to neural signal recording in multichannel systems. Epileptic signals are discussed from an engineering perspective, i.e., summarizing the major electrical characteristics and their impact on the microelectronics front-ends. The commercial implantable systems aiming at epilepsy control which command over an official approval at the moment of writing are reviewed. External systems which represent non-invasive systems are presented as a new trend in seizure alarming.

Circuit and system techniques are presented that aim at solving contemporary issues related to low-power, low-noise and low-area trade-offs met in modern microelectronics multi-channel neural acquisition systems. Specifically, the amplifier-sharing technique, as well as the compressed sensing technique, are discussed and presented.

## Abbreviations

The following abbreviations are used in this manuscript:

ACS	Analog Compressive Sensing
ADC	Analog-to-Digital Converter
AED	Anti-Epileptic Drug
AFE	Analog Front-End
BMI	Brain-Machine Interface
CC-LNA	Capacitive-Coupled Low-Noise Amplifier
CMRR	Common-Mode Rejection Ratio
CS	Compressive Sensing
CSF	Cerebrospinal Fluid
DBS	Deep-Brain Stimulation
DCS	Digital Compressive Sensing
DSL	dc Servo Loop
ECG	Electrocardiogram
ECoG	Electrocotricogram
EDA	Electrodermal Activity
EDO	Electrode dc Offset
EEG	Electroencephalogram
EMG	Electromyogram
ENOB	Effective Number of Bits
ERG	Electroretinogram
eTNS	External Trigeminal Nerve Stimulation
FDA	Food and Drug Administration
FDM	Frequency-Division Multiplexing
FE	Feature Extractor
FES	Functional Electrical Stimulation
ICP	Intracranial Pressure
iEEG	Intracranial Electroencephalography
IEMD	Implantable Electronic Medical Device
ILAE	International League Against Epilepsy
IMD	Implantable Medical Device
IPG	Integrated Pulse Generator
LFP	Local Field Potentials
LL	Line-length
LNA	Low-Noise Amplifier
MCS	Multi-channel Compressive Sensing
MEG	Magnetoencephalography
MISOCS	Multi-Input Single-Output Compressive Sensing
mMISOCS	modified Multi-Input Single-Output Compressive Sensing
MRI	Magnetic Resonance Imaging
NEF	Noise Efficiency Factor
OTA	Operational Transconductance Amplifier
PCG	Phonocardiogram
PET	Positron Emission Tomography
PGA	Programmable-Gain Amplifier
PMA	Premarket Approval
PPG	Photoplethysmogram
PSG	Polysomnography
RNS	Responsive Neurostimulation
PVT	Process, Voltage and Temperature
SEEG	Stereo-EEG
sEMG	Surface Electromyography
SNR	Signal-to-Noise Ratio
SPECT	Single-Photon Emission Computed Tomography
SUDEP	Sudden Unexpected Death in EPilepsy
tACS	transcranial Alternating Current Stimulation
tDCS	transcranial Direct Current Stimulation
TDM	Time-Division Multiplexing
tENS	transcutaneous Electric Nerve Stimulation
tTNS	Transcranial Trigeminal Nerve Stimulation
tVNS	transcutaneous Vagus Nerve Stimulation
VLSI	Very Large-Scale Integration
VNS	Vagus Nerve Stimulation
WHO	World Health Organization
